# Mechanistic Insights on the Mechanosynthesis of Phenytoin, a WHO Essential Medicine**

**DOI:** 10.1002/chem.202104409

**Published:** 2022-02-04

**Authors:** Francesco Puccetti, Stipe Lukin, Krunoslav Užarević, Evelina Colacino, Ivan Halasz, Carsten Bolm, José G. Hernández

**Affiliations:** ^1^ Institute of Organic Chemistry RWTH Aachen University Landoltweg 1 52074 Aachen Germany; ^2^ Division of Physical Chemistry Ruđer Bošković Institute Bijenička 54 10000 Zagreb Croatia; ^3^ Univ. Montpellier, CNRS, ENSCM, UM, ICGM Montpellier France

**Keywords:** ball milling, in situ monitoring, mechanochemistry, phenytoin, rearrangements

## Abstract

In recent years, mechanochemistry has enriched the toolbox of synthetic chemists, enabling faster and more sustainable access to new materials and existing products, including active pharmaceutical ingredients (APIs). However, molecular‐level understanding of most mechanochemical reactions remains limited, delaying the implementation of mechanochemistry in industrial applications. Herein, we have applied in situ monitoring by Raman spectroscopy to the mechanosynthesis of phenytoin, a World Health Organization (WHO) Essential Medicine, enabling the observation, isolation, and characterization of key molecular‐migration intermediates involved in the single‐step transformation of benzil, urea, and KOH into phenytoin. This work contributes to the elucidation of a reaction mechanism that has been subjected to a number of interpretations over time and paints a clear picture of how mechanosynthesis can be applied and optimized for the preparation of added‐value molecules.

## Graphical abstract

In situ monitoring by Raman spectroscopy of the mechanosynthesis of phenytoin, a World Health Organization Essential Medicine, is reported. This study enabled the observation, isolation and characterization of key molecular‐migration intermediates involved in the transformation of benzil (**1**), urea (**2**), and KOH into phenytoin (**3**). The depth of this investigation answers scientifically interesting questions, which might be helpful for the propulsion of mechanosynthetic protocols among mainstream methodologies for synthesis.

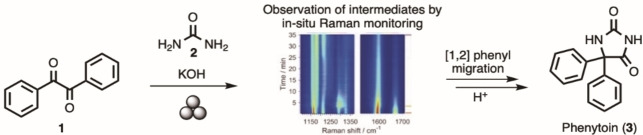

The simplicity, effectiveness and sustainability aspects of mechanochemistry^[1]^ have prompted its implementation for chemical synthesis across several fields of research.^[2]^ Some advantages of mechanosynthesis include complete or partial reduction of waste production, the acceleration of chemical transformations, and the opportunity to induce structural changes unattainable by other activation modes.^[3]^ With respect to applications, ball milling is undoubtedly the most versatile among the various techniques currently available in mechanosynthesis.^[4]^

Despite all the merits of mechanochemistry, the lack of mechanistic understanding of most mechanochemical transformations is delaying its application as a mainstream methodology for synthesis.^[5]^ A central challenge to study the evolution of chemical reactions in ball mills arises from the use of non‐transparent closed milling vessels operated at high speeds, which impedes the monitoring of the reactions using standard methods.^[6]^ However, over the past few years, this obstacle has been partially overcome with the development of in situ monitoring techniques based on synchrotron powder X‐ray diffraction and Raman spectroscopy,^[7]^ which have enabled a more detailed understanding of mechanochemical reactions by ball milling. For example, we recently studied the mechanochemical iconic benzil‐benzilic acid rearrangement, where in situ monitoring of the reaction by synchrotron X‐ray powder diffraction, Raman spectroscopy, and real‐time temperature sensing revealed the exact instant of the irreversible [1,2]‐intramolecular phenyl migration in benzil (**1**).^[9]^ Unfortunately, however, this approach also had limitations as none of the applied techniques allowed the detection of presumed intermediates before the rearrangement.^[10]^

Phenytoin (**3**) is a broadly prescribed anticonvulsant drug categorized as an essential medicine by the World Health Organization (WHO).^[11]^ The synthesis starts from benzil (**1**), urea (**2**), and KOH (Scheme 1), and, importantly, the reaction path shares mechanistic similarities with the previously studied benzil‐benzilic acid rearrangement,^[9]^ namely the involvement of an [1,2]‐intramolecular phenyl shift. Although this rearrangement has thoroughly been studied in solution,^[12]^ and preliminarily investigated by mechanochemistry,[^13^, ^14^] mechanistic details of the mechanochemical pathway providing phenytoin (**3**) have never been investigated. Thus, focusing on the phenytoin synthesis here had two attractive features: first, it was hoped that a concise analysis of the mechanochemical reaction path by continuous in situ methods^[15]^ would allow identifying intermediates and by‐products known to reduce the yield of the final product for the mechanochemical Biltz synthesis by ball milling.^[13]^ Second, and more generally, we aimed at demonstrating the applicability of mechanochemistry to access industrially relevant products.

**Scheme 1:**
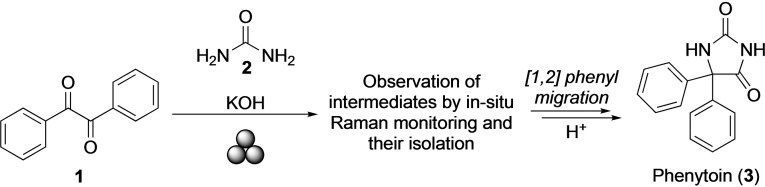
Mechanosynthesis of phenytoin (**3**). For mechanochemically activated reactions, the formalism proposed by Hanusa was used.^[8]^

In 1908, Heinrich Biltz reported the first synthesis of phenytoin (**3**) by heating an ethanolic alkaline solution of benzil (**1**) and urea (**2**).^[16]^ Ever since, studies in solution have demonstrated the importance of the reaction stoichiometry to favor the formation of phenytoin (**3**) over unwanted species such as the double condensation product 3a,6a‐diphenylglycoluril (**4**).^[12a]^ Therefore, our first task in investigating the mechanochemical route to phenytoin (**3**) consisted in establishing the optimal stoichiometric ratio for benzil (**1**) and urea (**2**) under solventless ball milling conditions (Figure 1a). For these experiments, the amount of KOH was set to two equivalents based on our previous studies.[^9^, ^13^] The results of this screening revealed that high conversion towards phenytoin (**3**) could be achieved by reacting equimolar amounts of benzil (**1**) and urea (**2**). In contrast the amount of by‐product **4** was kept low even in milling experiments using an excess of urea (Figure 1a). While some bases [K_2_CO_3_, Na_2_CO_3_, Ca(OH)_2_, and pyridine] did not lead to product formation, NaOH, KO*t*Bu and NaOEt were also found effective for the reaction (Figure 1b). However, the highest yield of phenytoin (**3**) was obtained with KOH (73 % after column chromatography). At least in part, these results might be due to rheological differences at the initial phase and changes during the proceeding of the reaction affecting the milling dynamics.

**Figure 1:**
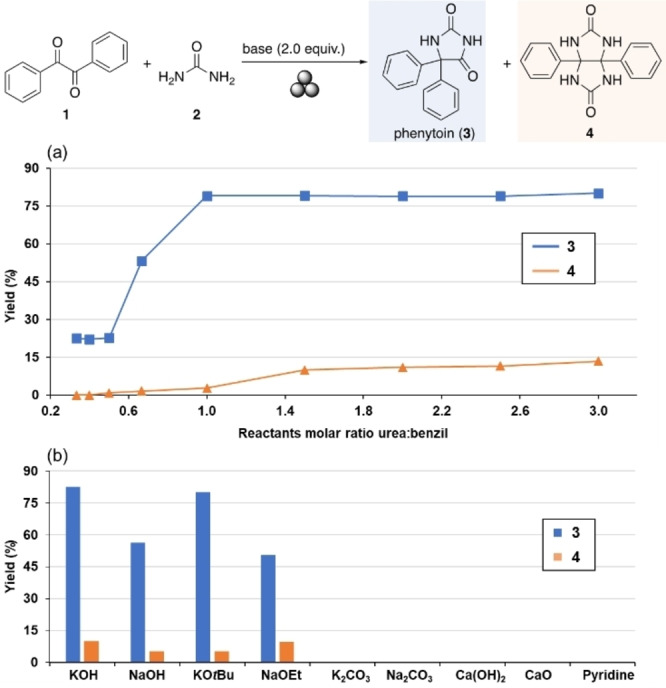
(a) Effect of the molar ratio of the reactants on the outcome of the reaction. Reaction conditions: **1** (0.30–0.90 mmol), **2** (0.30–0.90 mmol) and KOH (2.0 equiv.) were milled at 30 Hz for 90 min in a 5 mL stainless steel milling jar with one stainless steel milling ball of 7 mm in diameter (weighing 1.4 g). (b) Effect of the base on the formation of **3** by milling **1** (0.30 mmol) and **2** (0.45 mmol) at 30 Hz for 90 min. In all experiments, 0.60 mmol of the base was used except for Ca(OH)_2_ (0.30 mmol). Yields were determined by NMR spectroscopy using 1,3,5‐trimethoxybenzene as internal standard.

After identifying the experimental conditions to obtain phenytoin (**3**), its formation was studied during the ball milling process. For this, real‐time monitoring of the mechanochemical reaction between benzil (**1**), urea (**2**), and KOH was carried out by in situ Raman spectroscopy in transparent poly(methyl)methacrylate (PMMA) milling jars.^[17]^ In an initial monitoring experiment, an equimolar mixture of **1**, **2**, and KOH (2.0 equiv.) was milled at 30 Hz. In the first 5 min of milling, we observed a steady reduction in the intensity of several bands of **1** until the sudden loss of the Raman signal after ca. 5.5 min into milling (Figure 2a and Figures S1–S3 in Supporting Information). The loss of spectral information was due to rheological changes of the reaction mixture, which led to its compaction and sticking to the inner sidewall of the milling jar, preventing proper mixing and grinding. After an additional 4 min of milling, the reaction mixture became a free‐flowing powder again, and Raman spectra indicated the formation of the potassium salt of phenytoin **3**‐K as evidenced by the new bands at 1598 cm^−1^ and 1185 cm^−1^ (Figure 2a and Figures S1–S3). A detailed analysis of the Raman data revealed that, prior to the rheological changes in the reaction mixture, a new transient band that belongs to an intermediate phase was formed at around 1300 cm^−1^. The appearance of this new signal was accompanied by a slight, but distinctive, shift of the C−C stretching band of the phenyl rings^[18]^ of benzil (**1**) at 1594 cm^−1^ towards 1598 cm^−1^, in combination with the disappearance of other benzil bands (Figure 2a and Figures S1–S3).

**Figure 2:**
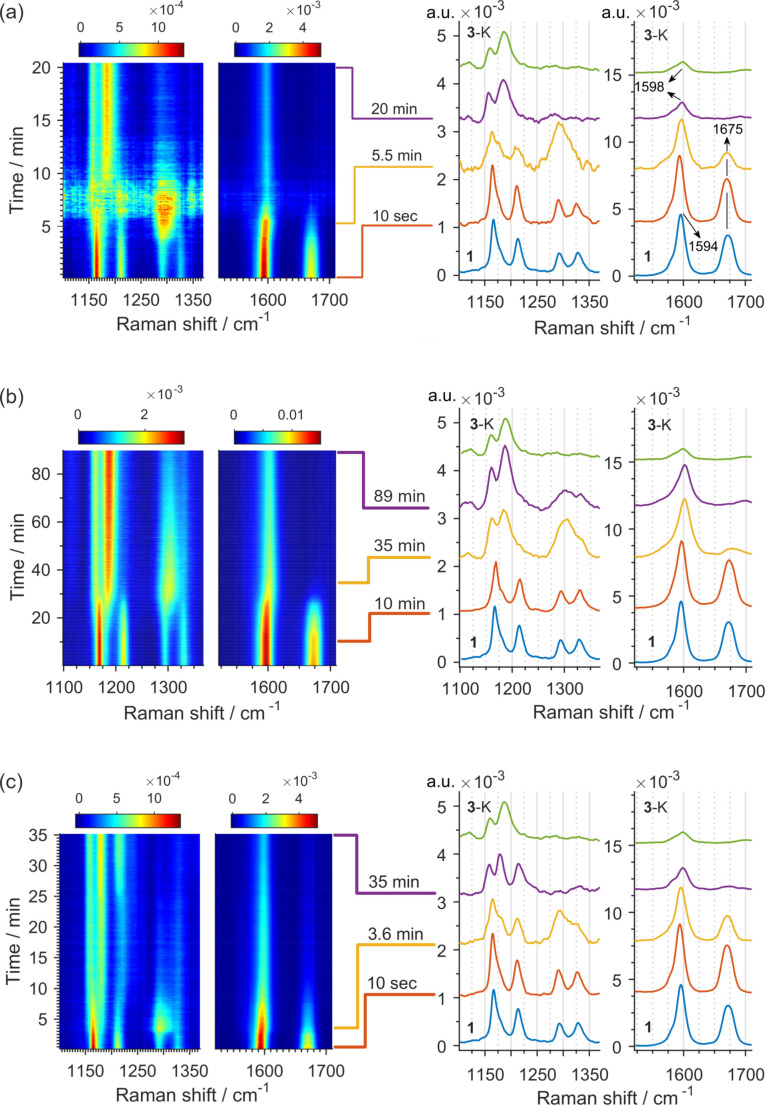
Time‐resolved 2D plots of the mechanochemical milling at 30 Hz of: (a) benzil:urea:KOH in 1 : 1 : 2 stoichiometric ratio and (right) Raman spectra of pure benzil (**1** – blue) at the bottom and potassium phenytoin salt (**3**‐K – dark green) at the top and selected spectra after 10 seconds, 5.5 min and 20 min of milling. (b) benzil:potassium ureate in 1 : 1 stoichiometric ratio (c) benzil:urea:KOH in 1 : 1 : 1 stoichiometric ratio.

The generally accepted mechanism for the synthesis of phenytoin considers the intermediacy of potassium ureate (**A**),^[12b]^ a stronger nucleophile than urea (Scheme 2a). To rule out a direct reaction between benzil and urea or the formation of a cocrystal between them, we first carried out the grinding of **1** and **2** for two hours. Monitoring by Raman spectroscopy revealed only the physical mixture of the starting materials (Figure S4), thus demonstrating that under mechanochemical conditions, urea is not sufficiently nucleophilic on its own and that the presence of KOH is necessary to form the actual nucleophilic intermediate **A**.

**Scheme 2:**
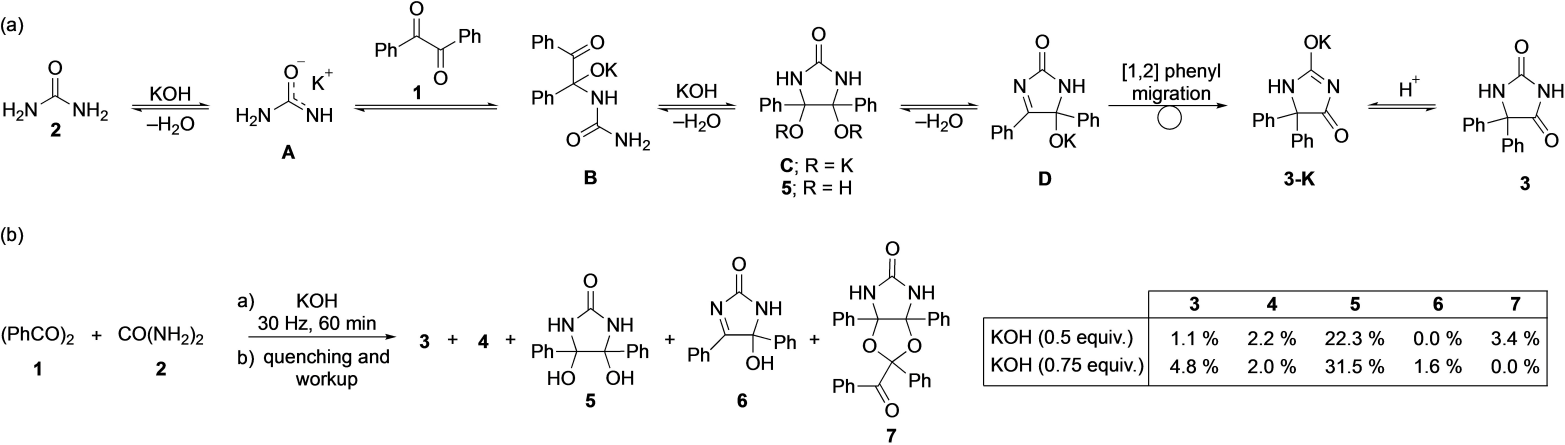
(a) Plausible mechanism for the formation of phenytoin (**3**) by ball milling. (b) Product distribution of the reaction **1**, **2** and KOH (0.5–0.75 equiv.). Yields were determined by NMR spectroscopy using 1,3,5‐trimethoxybenzene as internal standard.

Consequently, the release of water in the formation of **A** could have led to the rheological changes observed in the reaction mixture. Indeed, when urea was milled only with KOH, water droplets were observed on the milling jar wall while the reaction mixture fully stuck to one side of the milling jar wall. The use of milling‐neutral auxiliaries in the reaction of **1**, **2** and KOH (sand, KCl, neutral Al_2_O_3_ or ZrO_2_) could not prevent compacting and rheological changes of the reaction mixture. However, the formation of the phenytoin potassium salt **3**‐K was still achieved. The inclusion of water (2.0 equiv.) to the initial reaction mixture of **1**, **2** and KOH further favored the reactants’ agglomeration inside the milling jar. However, after 10 min of milling, the reaction mixture again became powdery, and **3** could be isolated in 60 % yield. These milling experiments indicate that the initial changes in rheology of the reaction mixture (i. e., stickiness and compaction of the sample) are transitory and do not inhibit the formation of **3**, which is achievable even in the presence of water.

Once potassium ureate (**A**) is formed, it can act as a nucleophile towards one carbonyl group of **1**, affording intermediate species such as **B** (Scheme 2a). From the continuous Raman monitoring of the reaction, we observed a correlation between the attenuation in the intensity of the band at 1675 cm^−1^, that corresponded to the C=O stretching in benzil, and the appearance of a new band at 1300 cm^−1^ (Figure 2 and Figures S1‐S3),^[18]^ which could correspond to the addition product **B** (Scheme 2a). Such a variation simultaneously proceeds with the previously described shift of the C−C stretching band of the phenyl rings (Figure 2a). Next, potassium ureate (**A**)^[19]^ was independently prepared, and its reaction with benzil (**1**) in the absence of the KOH was monitored in situ (Figure 2b). This experiment clearly showed the formation of the band at 1300 cm^−1^ and a gradual transformation to phenytoin during 90 min, indicating the intermediacy of potassium ureate (**A**) and **B** in the formation of phenytoin (**3**) by ball milling. This reaction could be accelerated in the presence of KOH (0.5 equiv.) (Figure S5).

In the search to identify additional intermediates involved in the formation of **3**, the reaction of **1** and **2** with smaller amounts of KOH (0.5–1.0 equiv.) was monitored. In the experiment using 1.0 equiv. of KOH the reaction mixture remained a free‐flowing powder throughout the entire experiment, and the real‐time Raman data revealed the formation of a new phase that did not correspond to the phenytoin potassium salt **3**‐K; similar results were obtained using 0.75 or 0.5 equiv. of KOH. This new species had characteristic bands at 1214 cm^−1^ and 361 cm^−1^, and persisted unchanged over 120 min of milling (Figure 2c and Figure S6–S8). Motivated by the freedom to maneuver that the real‐time monitoring provided us and thanks to the apparent stability of the newly detected phase, we attempted to isolate it. For this, **1**, **2**, and KOH (0.75 or 0.5 equiv.) were reacted for 60 min followed by the quenching of the reaction mixture. Analysis of the crude mixture by NMR spectroscopy showed unreacted starting materials and traces of **3** and **4**. More importantly, diol **5** was detected as the major component of the mixture along with minor amounts of products **6** and **7** (Scheme 2b). We surmised here that both diol **5** and imidazolone **6** reasonably relate to the corresponding intermediates **C** and **D**. The identification of **5** and **6** was confirmed after their independent synthesis (for details, see the Supporting Information). At the same time, ketal **7** was isolated from the mechanochemical reaction mixture. Independently, it was shown that **6** reacted with benzil and KOH providing **7**. The observation and posterior isolation of the diol **5** and the imidazolone **6** is particularly relevant, as they correspond to intermediates originally proposed in the formation of phenytoin in solution, but whose isolation proved difficult (for **5**) or not possible (for **6**).^[12]^ Conclusively, when the independently synthesized diol **5** or 1,5‐dihydro‐2*H*‐imidazol‐2‐one **6** were milled with KOH, they readily rearranged into **3**, thus confirming their likely intermediacy in the reaction. The formation of **6** from the diol **5** was additionally corroborated by monitoring the spontaneous dehydration of **5** in solution by NMR spectroscopy (Figure S13). The observation of **6** in traces in the crude mixture (Scheme 2b) indicates that the phenytoin potassium salt **3**‐K could have formed via the intermediate **D**, although the presence of **D** was not detected during the in situ Raman monitoring. In fact, the formation of **3**‐K from diol **5** through the dehydration and the irreversible [1,2]‐intramolecular phenyl migration is expected to occur rapidly, as previously demonstrated for the analogous benzilic acid rearrangement.^[9]^

In summary, we performed a detailed mechanistic analysis of the mechanosynthesis of phenytoin, a WHO Essential Medicine. We clarified the importance of the stoichiometry of the reactants and the role of different bases ex situ initially. Then, the application of in situ Raman monitoring techniques enabled unprecedented real‐time insight into the mechanosynthetic pathway and allowed for the observation and isolation of elusive intermediates such as diol **5** and imidazolone **6**, as well as unprecedented observation of ketal **7**. Intermediates **5** and **6** had previously been proposed for conventional synthesis but were hard or impossible to isolate from the solution. From a more general perspective, the depth of the reported investigation on the study of the mechanosynthesis of the phenytoin API answers scientifically interesting questions, which might be helpful for the propulsion of mechanosynthetic protocols among mainstream methodologies for synthesis.

## Conflict of interest

The authors declare no conflict of interest.

## Supporting information

As a service to our authors and readers, this journal provides supporting information supplied by the authors. Such materials are peer reviewed and may be re‐organized for online delivery, but are not copy‐edited or typeset. Technical support issues arising from supporting information (other than missing files) should be addressed to the authors.

Supporting Information

## Data Availability

The data that support the findings of this study are available in the supplementary material of this article.
