# Supplementary material for: Mechanistic Insights on the Mechanosynthesis of Phenytoin, a WHO Essential Medicine
Source: Chemistry. 2022 Feb 4;28(13):e202104409. doi: 10.1002/chem.202104409 (PMC9304275; doi:10.1002/chem.202104409)
Supplement: Supplementary file 1 — Supporting Information [file CHEM-28-0-s001.pdf]

# Chemistry–A European Journal

Supporting Information


## Mechanistic Insights on the Mechanosynthesis of Phenytoin, a WHO Essential Medicine


Francesco Puccetti, Stipe Lukin, Krunoslav Užarević, Evelina Colacino, Ivan Halasz,* Carsten Bolm,* and José G. Hernández*

## Table of Contents

**1. General information** S4

**1.1 Chemicals** S4

**1.2 Milling equipment** S4

**1.3 Nuclear Magnetic Resonance (NMR) spectroscopy** S4

**1.4 Infrared spectroscopy (IR)** S4

**1.5 Melting point (m.p.)** S4

**1.6 Thin layer chromatography (TLC) and column chromatography** S4

**1.7 Mass spectrometry (MS)** S4

**1.8 Quantitative $^{13}$C NMR spectroscopy experiments** S5

**1.9 Mechanosynthesis of phenytoin (3); optimization of molar ratios of the reactants** S5

**1.10 In-situ monitoring of the mechanosynthesis of phenytoin by Raman spectroscopy** S6

**2. Additional figures** S7

**Figure S1-S11** – Additional Raman spectra and time-resolved 2D plots S7-S12

**Figure S12** – Quantitative $^{13}$C NMR analysis for reaction optimization S13

**Figure S13** – Dehydration of **5** into **6** monitored by $^{1}$H NMR spectroscopy S14

**3. Experimental synthetic procedures** S15

**3.1 Phenytoin (3)** S15

**3.2 3a,6a-Diphenylglycoluril (4)** S15

**3.3 4,5-Diphenyl-4-imidazolin-2-one (S1)** S17

**3.4 5-Hydroxy-4,5-diphenyl-1,5-dihydro-2*H*-imidazol-2-one (6)** S118

**3.5 4,5-Dihydroxy-4,5-diphenyl-imidazolidin-2-one (5)** S20

**3.6 2-Benzoyl-2,3a,6a-triphenyltetrahydro-5*H*-[1,3]dioxolo[4,5-*d*]imidazol-5-one (7)** S21

**3.7 Phenytoin potassium salt (3-K)** S22

**3.8 Potassium ureate (A)** S23

**4. Additional control reactions** S24

**4.1 Reaction of oven dried benzil (1), urea (2) and KOH under inert argon atmosphere** S24

**4.2 Reaction of benzil (1), urea (2), KOH and $H_2O$** S24

**4.3 Reaction of 4,5-dihydroxy-4,5-diphenyl-imidazolidin-2-one (5) + KOH** S24

**4.4 Reaction of 5-hydroxy-4,5-diphenyl-1,5-dihydro-2*H*-imidazol-2-one (6) + KOH** S24

**4.5 Reaction of 5-hydroxy-4,5-diphenyl-1,5-dihydro-2*H*-imidazol-2-one (6) + urea (2) + KOH** S24

**4.6 Reaction of 5-hydroxy-4,5-diphenyl-1,5-dihydro-2*H*-imidazol-2-one (6) + benzil (1) + KOH** S24

**4.7 Variations from the reaction conditions reported in Section 3.6** S25

**5. References** S26

**6. Copies of the NMR spectra** S27

**Figure S14 –** $^{1}$H NMR (600 MHz, DMSO-$d_6$) spectrum of **3**. S27

**Figure S15 –** $^{13}$C{$^{1}$H} NMR (150 MHz, DMSO-$d_6$) spectra of **3**. S27

**Figure S16 –** $^{1}$H NMR (600 MHz, DMSO-$d_6$) spectrum of **4**. S28

**Figure S17 –** $^{13}$C{$^{1}$H} NMR (150 MHz, DMSO-$d_6$) spectra of **4**. S28

**Figure S18 –** $^{1}$H NMR (400 MHz, DMSO-$d_6$) spectrum of **S1**. S29

**Figure S19 –** $^{13}$C{$^{1}$H} NMR (101 MHz, DMSO-$d_6$) spectra of **S1**. S29

**Figure S20 –** $^{1}$H NMR (600 MHz, DMSO-$d_6$) spectrum of **6.** S30

**Figure S21 –** $^{13}$C{$^{1}$H} NMR (150 MHz, DMSO-$d_6$) spectra of **6.** S30

**Figure S22 –** $^{1}$H,$^{13}$C-HSQC NMR (600,150 MHz, DMSO-$d_6$) spectrum of **6.** S31

**Figure S23 –** $^{1}$H NMR (600 MHz, DMSO-$d_6$) spectrum of **5.** S32

**Figure S24 –** $^{13}$C{$^{1}$H} NMR (150 MHz, DMSO-$d_6$) spectra of **5.** S32

**Figure S25 –** $^{1}$H NMR (600 MHz, DMSO-$d_6$) spectrum of **7.** S33

**Figure S26 –** $^{13}$C{$^{1}$H} NMR (150 MHz, DMSO-$d_6$) spectra of **7.** S33

**Figure S27 –** $^{1}$H,$^{1}$H-TOCSY NMR (600,600 MHz, DMSO-$d_6$) spectrum of **7.** S34

**Figure S28 –** $^{1}$H NMR (600 MHz, $D_2O$) spectra of **3**-K**.** S35

**Figure S29 –** $^{13}$C{$^{1}$H} NMR (150 MHz, $D_2O$) spectra of **3**-K**.** S35

**Figure S30 –** $^{1}$H NMR (600 MHz, DMSO-$d_6$) spectra of **A.** S36

**Figure S31 –** $^{13}$C{$^{1}$H} NMR (150 MHz, DMSO-$d_6$) spectra of **A.** S36

# 1. General information

### 1.1 Chemicals

Benzil (Acros), urea (BASF), potassium hydroxide (Grüssing), 5,5-diphenylhydantoin (Alfa Aesar), benzoin (Alfa Aesar), and all the mentioned chemicals in this document are commercially available and were used as received unless otherwise stated.

### 1.2 Milling equipment

Optimization of the mechanochemical reactions was carried out using a Retsch MM400 Mixer Mill using milling jars made of stainless steel (SS) (5.0 mL in internal volume) and one milling ball made of stainless steel (7.0 mm in diameter, weighing 1.4 g). The ball mill was operated at a frequency of 30 Hz.
Mechanochemical monitoring reactions were carried out using an IST-636 mixer mill (InSolido Technologies, Zagreb, Croatia) using milling jars made of poly(methyl)methacrylate (PMMA) (15 mL in internal volume) and one milling ball made of stainless steel (10.0 mm in diameter, weighing 4.0 g). The ball mill was operated at a frequency of 30 Hz.

### 1.3 Nuclear Magnetic Resonance (NMR) spectroscopy

Nuclear magnetic resonance (NMR) spectra were recorded either on a Varian Mercury 300 MHz, Varian VNMRS 400 MHz, Varian VNMRS 600 MHz, Bruker Avance Neo 400 MHz or Bruker Avance Neo 600 MHz spectrometers at 25 °C, if not otherwise stated and were processed and analyzed with the program MestReNova.[1] The chemical shifts $\delta$ are given in parts per million (ppm) relative to the residual solvent peak of the non-deuterated solvent (DMSO-$d_6$: $^1$H NMR: $\delta$ = 2.50 ppm, $^{13}$C{$^1$H}NMR: $\delta$ = 39.52 ppm or $D_2O$: $^1$H NMR: $\delta$ = 4.79 ppm).[2] Carbon spectra were measured broad band decoupled, if not otherwise stated. The multiplicity was reported with the following abbreviations: s = singlet, d = doublet, t = triplet, q = quartet, m = multiplet, br = broad signal and combinations thereof. Coupling constants (*J*) were given in Hertz (Hz).

### 1.4 Infrared spectroscopy (IR)

Infrared spectra (IR) were recorded as transmission spectra on a PerkinElmer Spectrum 100 spectrometer using the attenuated total reflectance (ATR) technique. The wavenumbers $\nu$ of the absorption peaks are listed in cm$^{-1}$.

### 1.5 Melting point (m.p.)

Melting points were measured with a Büchi Melting Point M-560 machine.

### 1.6 Thin layer chromatography (TLC) and column chromatography

Thin-layer chromatography (TLC) was performed on TLC plates (silica gel 60 on aluminum plates with fluorescence indicator F254) from MERCK. Qualitative analysis of the TLC plates was carried out using UV light ($\lambda$ = 254 nm and/or $\lambda$ = 366 nm) and/or by immersion in TLC stain solutions of potassium permanganate ($KMnO_4$) or vanillin.
Flash column chromatography was conducted on silica gel 60 (40−63 $\mu$m) from MACHEREY-NAGEL and solvents for column chromatography were distilled prior to use.

### 1.7 Mass spectrometry (MS)

Mass spectra were recorded on a Finnigan SSQ 7000 spectrometer [electron ionization (EI): 70 eV; chemical ionization (CI): methane, 100 eV] and the resulting signals are given according to their *m/z* values and their relative intensity is reported in parentheses. For atmospheric pressure chemical ionization mass spectra (APCI), a Bruker maXis II UHR-TOF LC-MS-System (Ion-Source: APCI-Source) was used. For high resolution mass spectra (HRMS) a Thermo Fisher Scientific LTQ Orbitrap XL spectrometer [electrospray ionization (ESI) in positive ion mode] was used.

### 1.8 Quantitative $^{13}$C NMR spectroscopy experiments

The experiments were conducted on a Varian VNMRS 600 MHz spectrometer. The spectra were recorded at 25°C in inverse gated decoupling mode using 512 scans and a relaxation delay of 15.0 seconds. To evaluate the actual efficacy of the method, the integrals of the $^{13}$C signals of the internal standard 1,3,5-trimethoxybenzene in a DMSO-$d_6$ have been used as benchmark. In the picture below are reported the integrals values for the same sample analyzed in (a) the usual $^{13}$C{$^1$H} full decoupled mode and (b) the $^{13}$C{$^1$H} prolonged relaxation gated decoupling mode. As expected, the same integral value for the three peaks is observed (within the limits of a variation of ±1%).

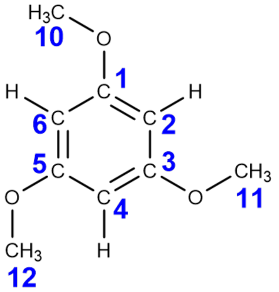


(a)

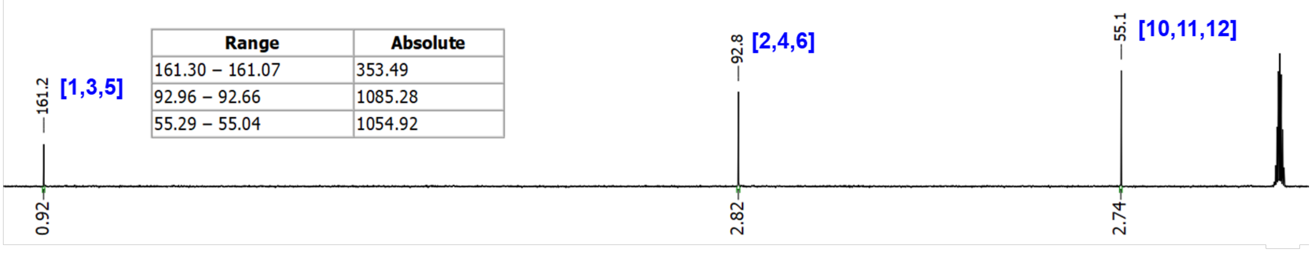


(b)

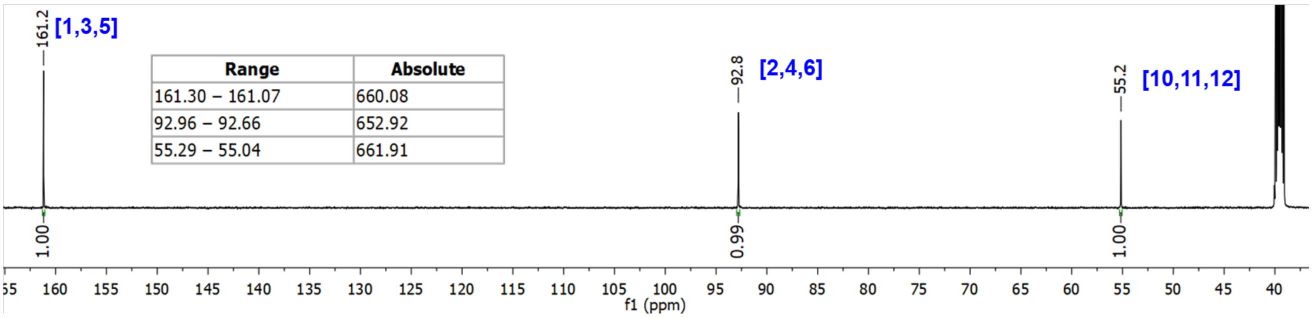


### 1.9 Mechanosynthesis of phenytoin (3); optimization of molar ratios of the reactants

Reaction conditions

1. Take a 5.0 mL stainless steel milling jar and equip it with one 7 mm stainless steel (weighing 1.4 g) milling ball.
2. In open air, add in sequence to the jar: benzil (**1**, 63.1-184.0 mg, 0.3-0.9 mmol), urea (**2**, 18.0-54.1 mg, 0.3-0.9 mmol) and KOH (33.7 mg, 0.6 mmol).
3. Mill the reaction mixture at 30 Hz for 90 minutes.

Extraction and analysis

4. Open the milling jar, add $NH_4Cl$ (33.7 mg, 0.63 mmol) and 1.0 mL of AcOEt.
5. Mill the mixture at 20 Hz for 5 minutes.
6. Open the jar and transfer the slurry to a round bottom flask with the help of ca. 10 mL of AcOEt.
7. Evaporate the organic solvent under vacuum and dry the residual solid mixture under high vacuum for 30 minutes.
8. Add the NMR internal standard (1,3,5-trimethoxybenzene) directly to the dried mixture in the flask.
9. Dissolve the solids DMSO-$d_6$ and filtrate on celite directly in an NMR tube.
10. Record the NMR spectra of the mixture using the quantitative $^{13}$C NMR method (see section 1.8).

**1.10 In-situ monitoring of the mechanosynthesis of phenytoin by Raman spectroscopy**

Laboratory in-situ Raman monitoring was performed using portable Raman system with a PDLD (now Necsel) BlueBox laser source with the excitation wavelength of 785 nm equipped with B&W-Tek fiber optic Raman BAC102 probe and coupled with one of OceanOptics Maya2000Pro spectrometers (with resolutions of 1 $cm^{-1}$ or 3.5 $cm^{-1}$). The probe was positioned about 1 cm under a reaction vessel on a moving stand, and laser was focused 1 mm inside of the inner vessel wall. Time-resolved in-situ Raman spectra were collected in an automated fashion using an in-house code in MATLAB. Subtraction of vessel contribution to Raman spectra was described elsewhere.[3] Raman spectra of pure reactants and products were baseline corrected using asymmetric least-squares algorithm[4] and subsequently normalized with Euclidean (L1) norm.

Each collected Raman spectrum was cut to fit into spectral range from 40 – 1710 $cm^{-1}$. After baseline correction with the asymmetric least-squares,[3,4] the spectrum was soothed using Withaker smoother[5] and normalized using L1 norm. The part of spectrum without the signal was left out from the 2D plots.

## 2. Additional figures

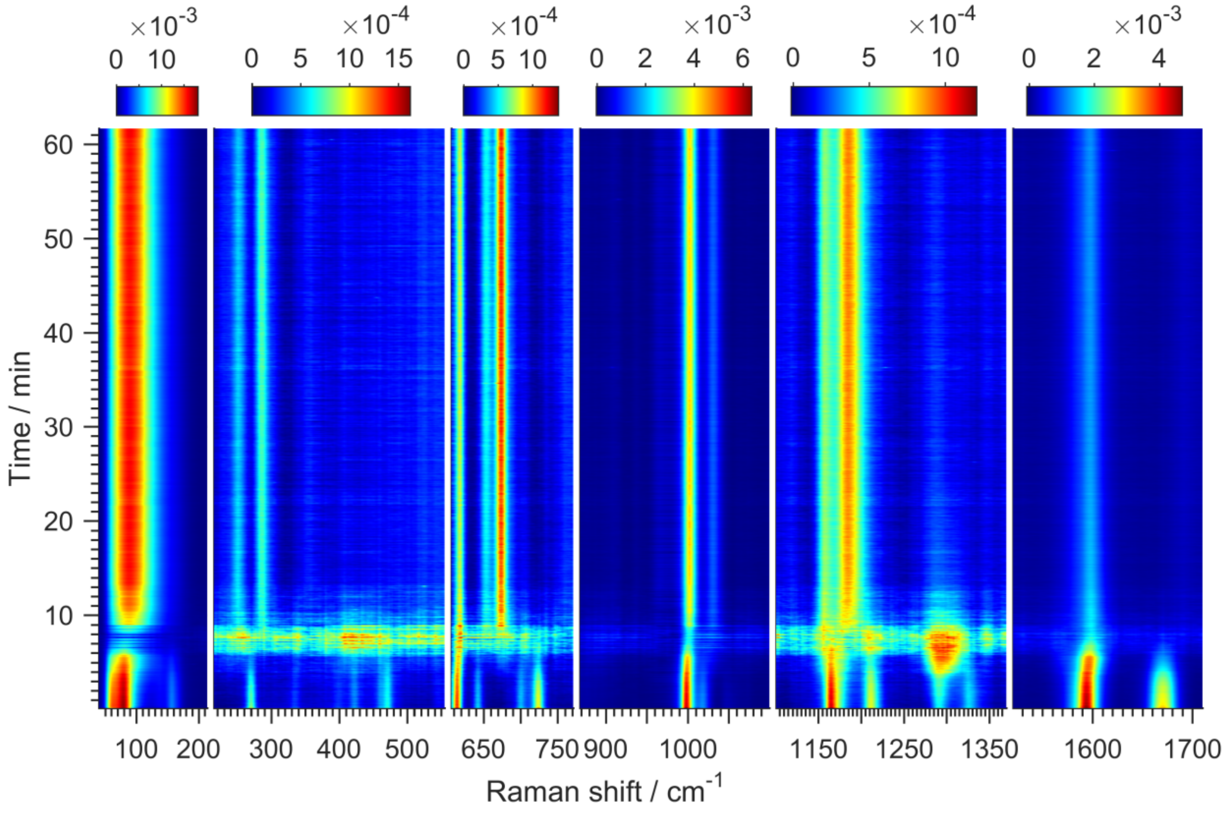


**Figure S1**. Time-resolved 2D plot of mechanochemical milling of benzil:urea:KOH in a 1:1:2 stoichiometric ratio. The plot contains selected parts of the whole spectral range that differ in intensity.

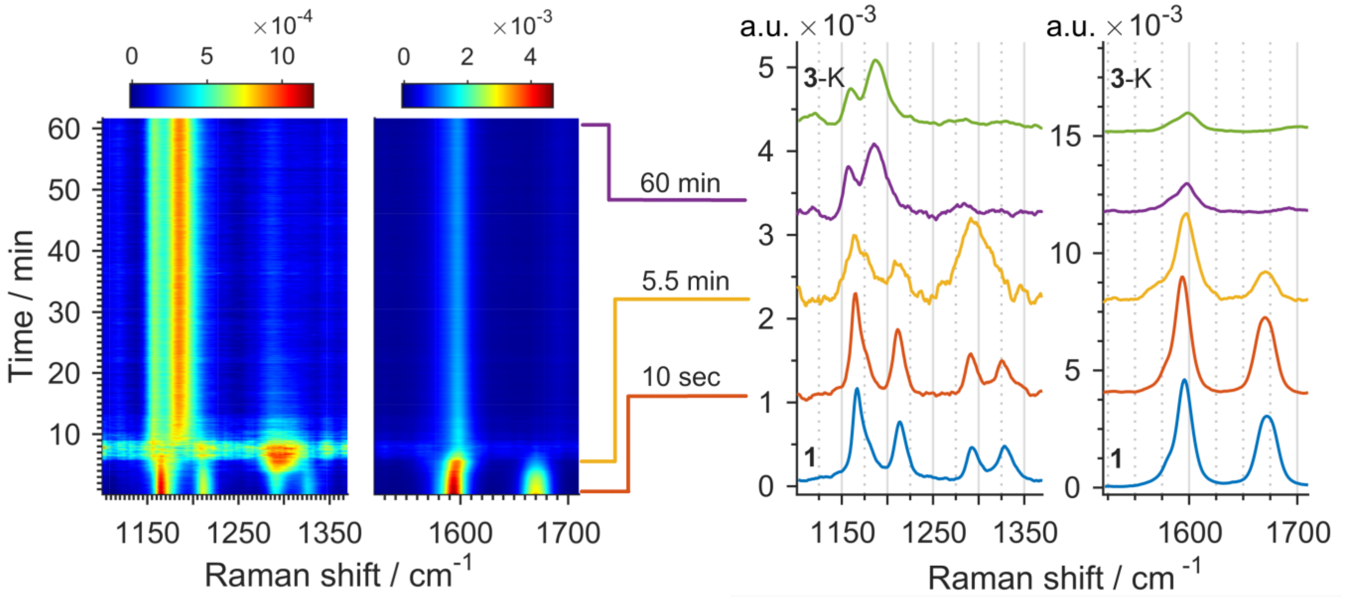


**Figure S2**. Time-resolved 2D plot of mechanochemical milling of benzil:urea:KOH in a 1:1:2 stoichiometric ratio (left) and confrontation of Raman spectra of pure benzil (**1** - blue) on the bottom, potassium phenytoin salt (**3**-K - dark green) at the top and selected spectra after 10 seconds, 5.5 minutes and 60 minutes of milling (right).

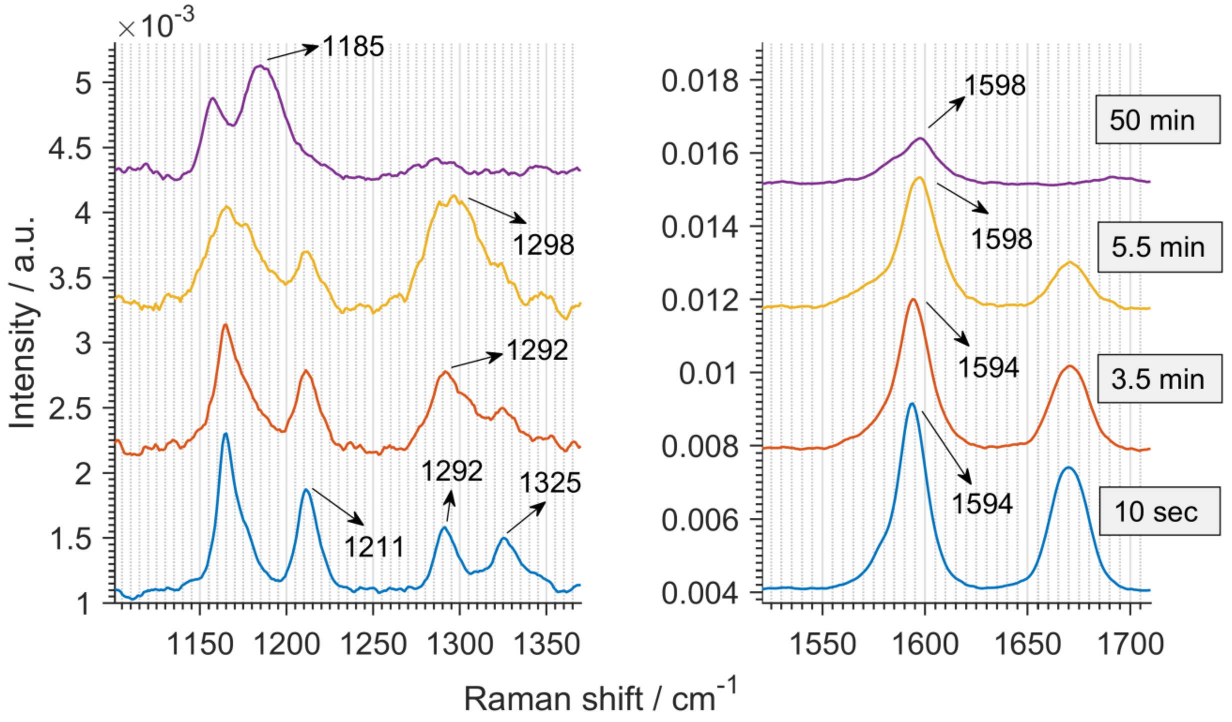


**Figure S3**. Stacked Raman spectra after 10 seconds, 3.5 min, 5.5 min, and 50 min into the milling of benzil:urea:KOH in a 1:1:2 stoichiometric ratio. The disappearance and the formation of Raman bands are designated. The band forming at 1298 cm$^{-1}$ belongs to an intermediate. The strong benzil band at 1594 cm$^{-1}$ corresponds to the aromatic ring breathing vibrations, shifting towards higher energies as the reaction proceed and to potassium phenytoin at 1598 cm$^{-1}$.

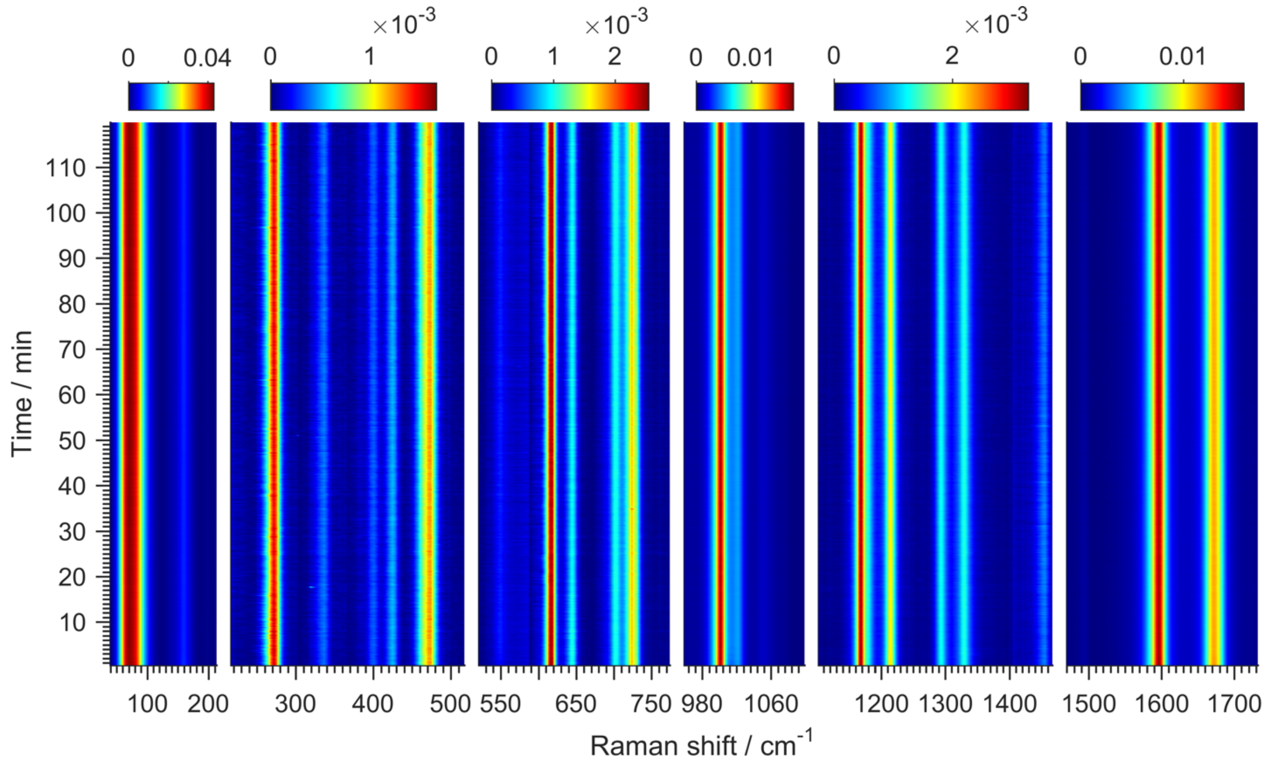


**Figure S4.** Time-resolved 2D plot of mechanochemical milling of benzil:urea in a 1:1 stoichiometric ratio. The plot contains selected parts of the whole spectral range that differ in intensity.

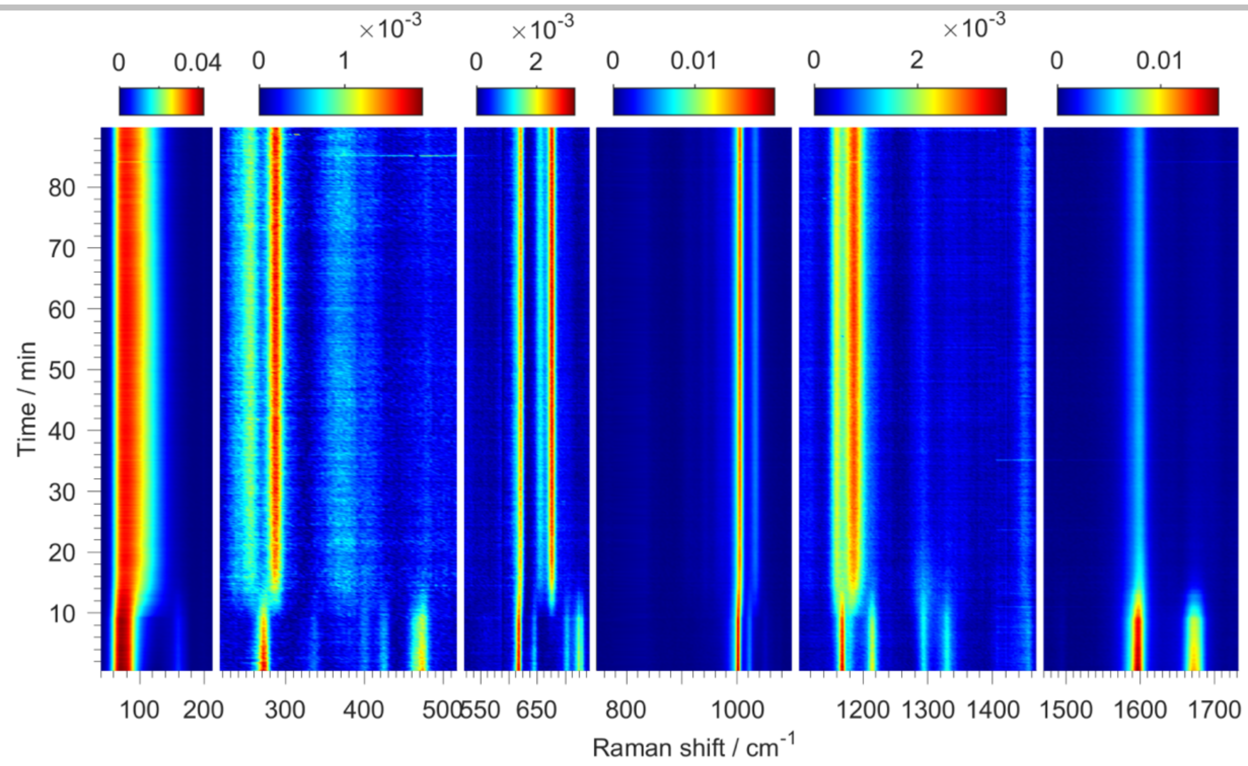


**Figure S5**. Time-resolved 2D plot of mechanochemical milling at 30 Hz of benzil:potassium ureate:KOH in a 1:1:0.5 stoichiometric ratio. Plot contains selected parts of the whole spectral range that differ in intensity.

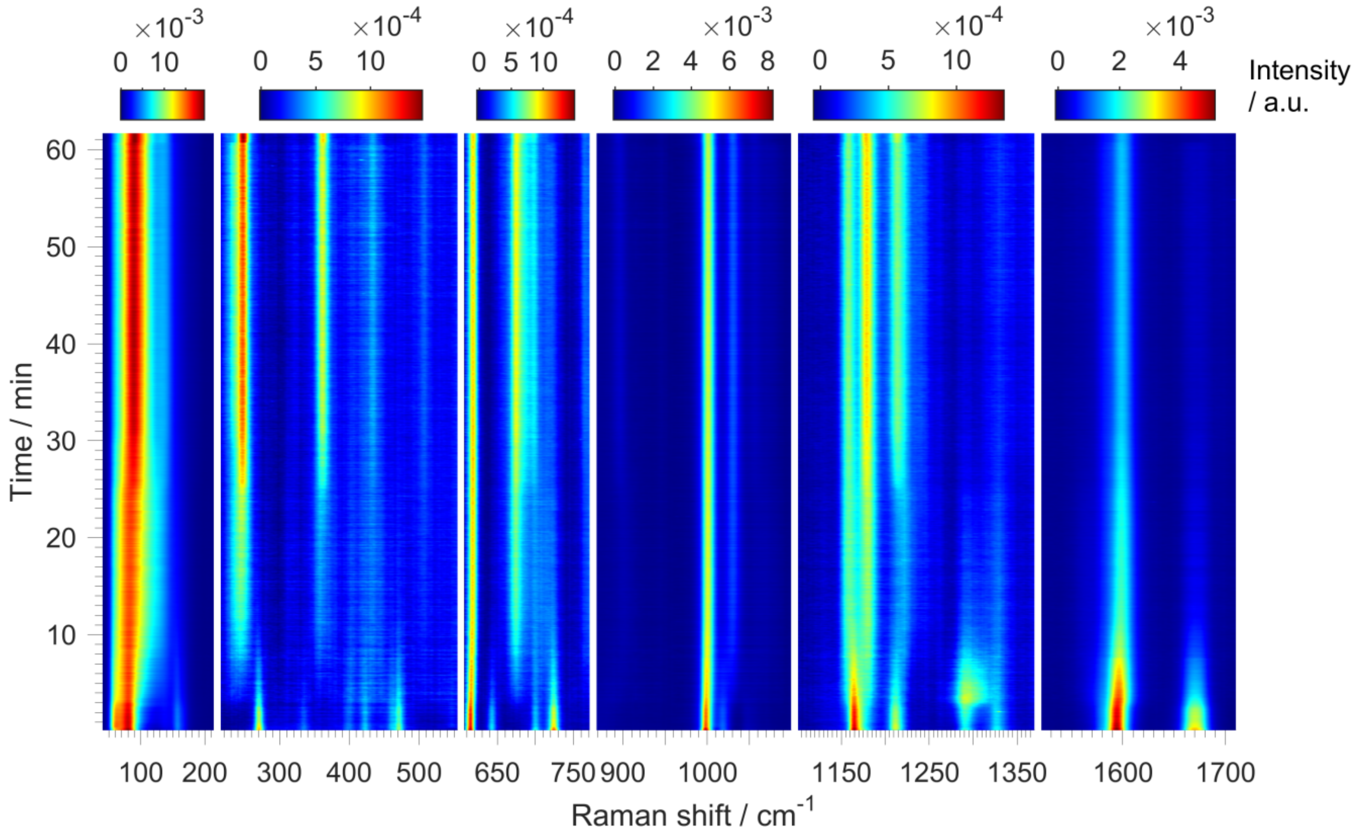


**Figure S6**. Time-resolved 2D plot of mechanochemical milling of benzil:urea:KOH in a 1:1:1 stoichiometric ratio. The plot contains selected parts of the whole spectral range that differ in intensity

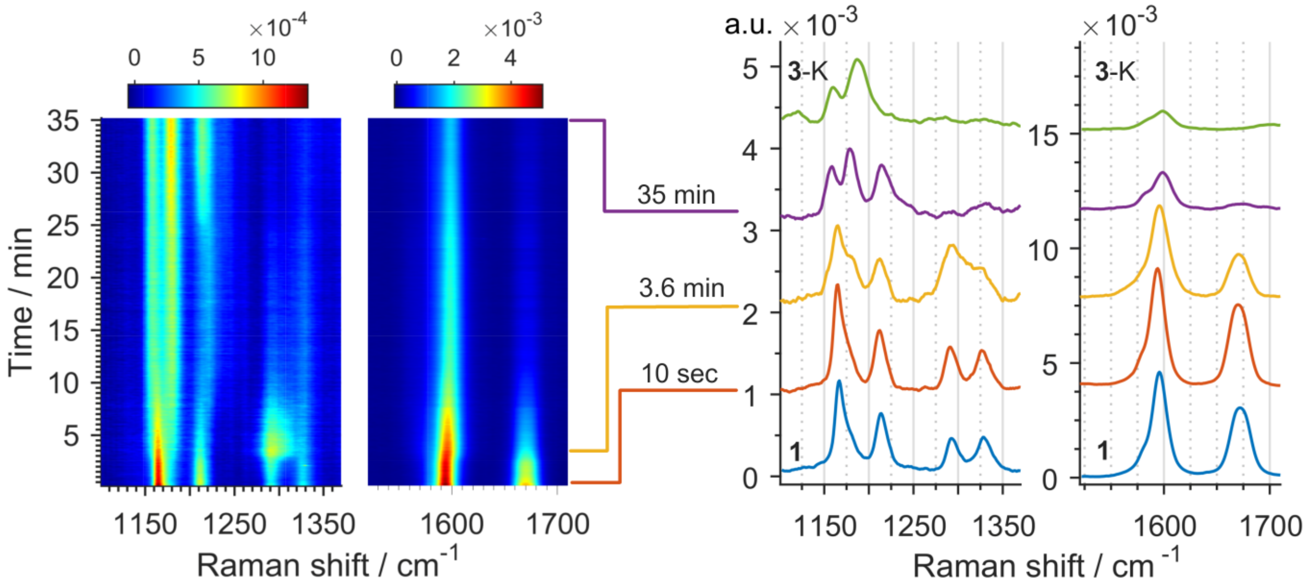


**Figure S7**. Time-resolved 2D plot of mechanochemical milling of benzil:urea:KOH in a 1:1:1 stoichiometric ratio (left) and confrontation of Raman spectra of pure benzil (**1** - blue) on the bottom, potassium phenytoine salt (**3**-K – dark green) at the top and selected spectra after 10 seconds, 3.6 minutes and 35 minutes of milling (right).

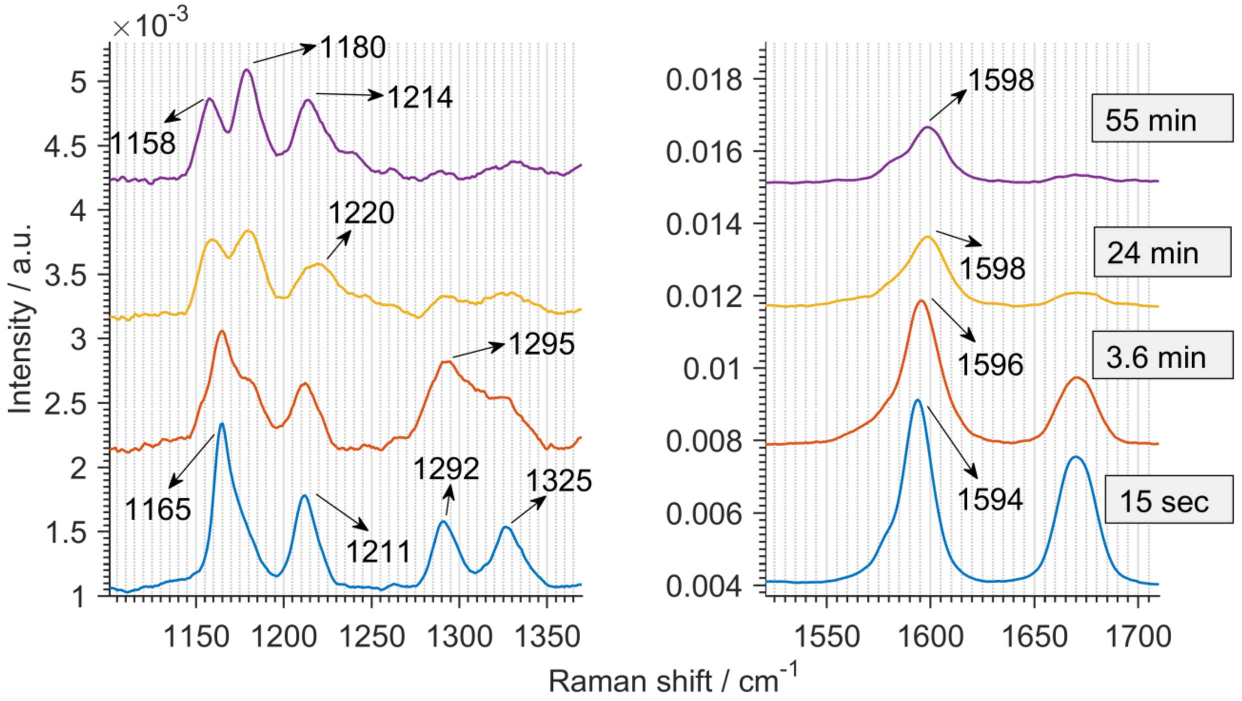


**Figure S8**. Stacked Raman spectra after 15 seconds, 3.6 min, 24 min, and 55 min into the milling of benzil:urea:KOH in 1:1:1 stoichiometric ratio. The disappearance and the formation of Raman bands are designated. The band forming at 1295 cm$^{-1}$ belongs to an intermediate. The formation of the band at 1214 cm$^{-1}$ indicates the presence of additional phases in the final reaction mixture. The strong benzil band at 1594 cm$^{-1}$ corresponds to the aromatic ring breathing vibrations, shifting towards higher energies as the reaction proceed and to potassium phenytoin at 1598 cm$^{-1}$.

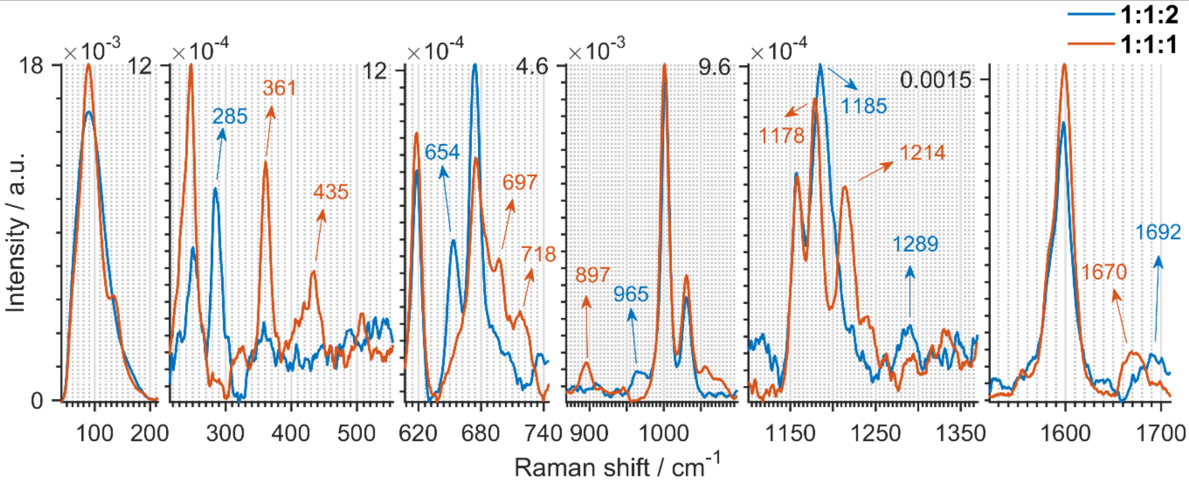


**Figure S9**. Comparison of the selected spectral ranges of Raman spectra of the reaction mixture after 55 minutes of milling for 1:1:2 and 1:1:1 stoichiometry. In 1:1:2 case, the Raman spectrum corresponds to the potassium phenytoin, while in the 1:1:1 case additional phases are present.

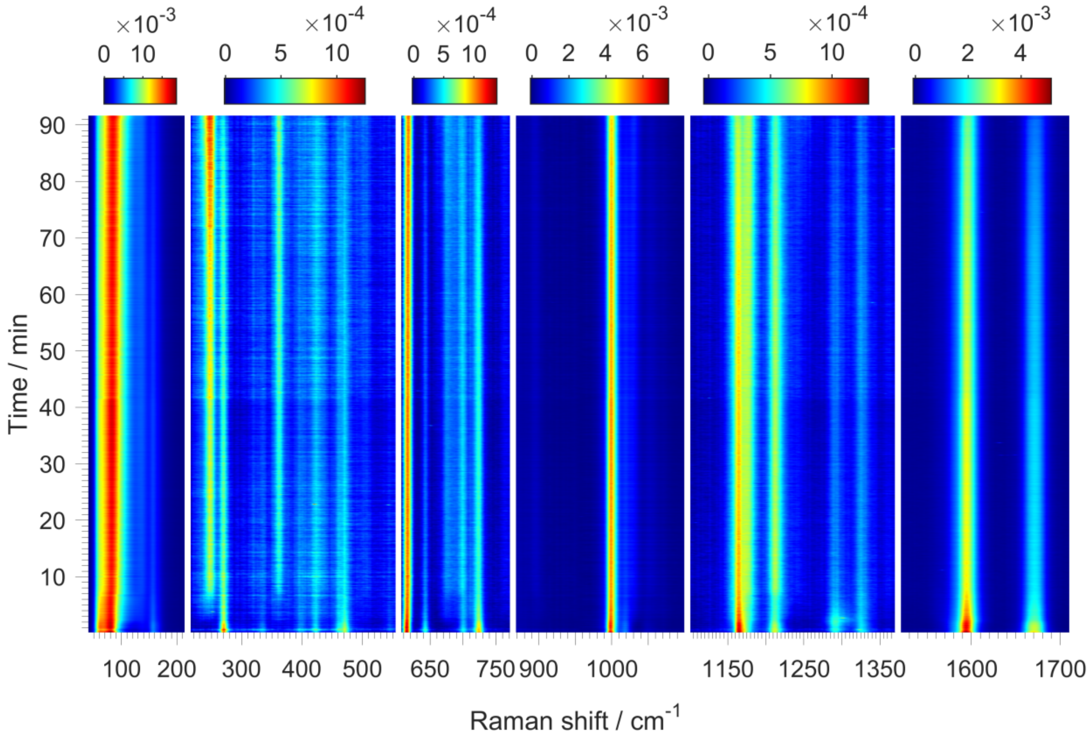


**Figure S10.** Time-resolved 2D plot of mechanochemical milling of benzil:urea:KOH in a 1:1:0.5 stoichiometric ratio. The plot contains selected parts of the whole spectral range that differ in intensity.

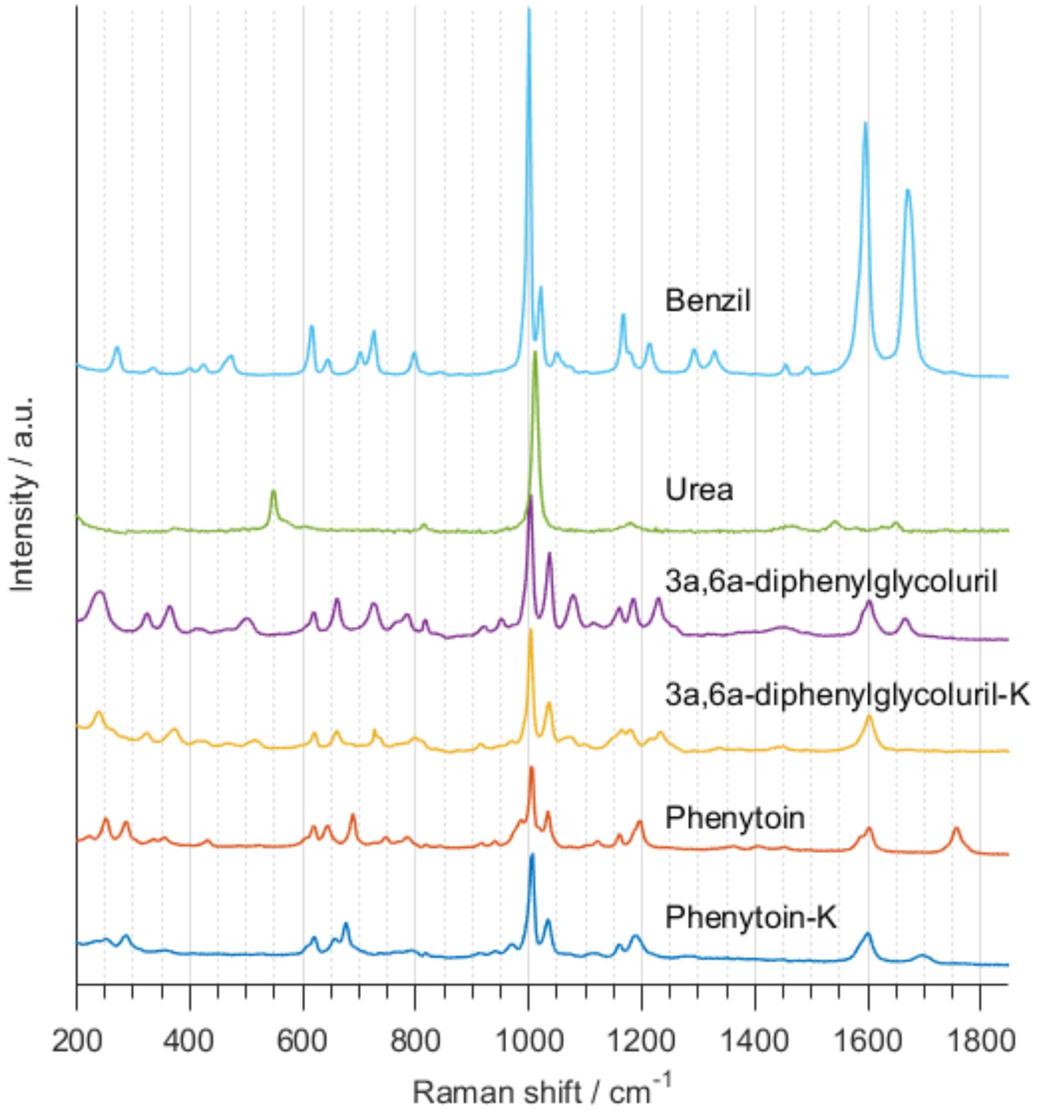


**Figure S11**. Stacked Raman spectra of pure benzil, urea, 3a,6a-diphenylglycoluril, 3a,6a-diphenylglycoluril potassium salt, phenytoin, and phenytoin potassium salt.

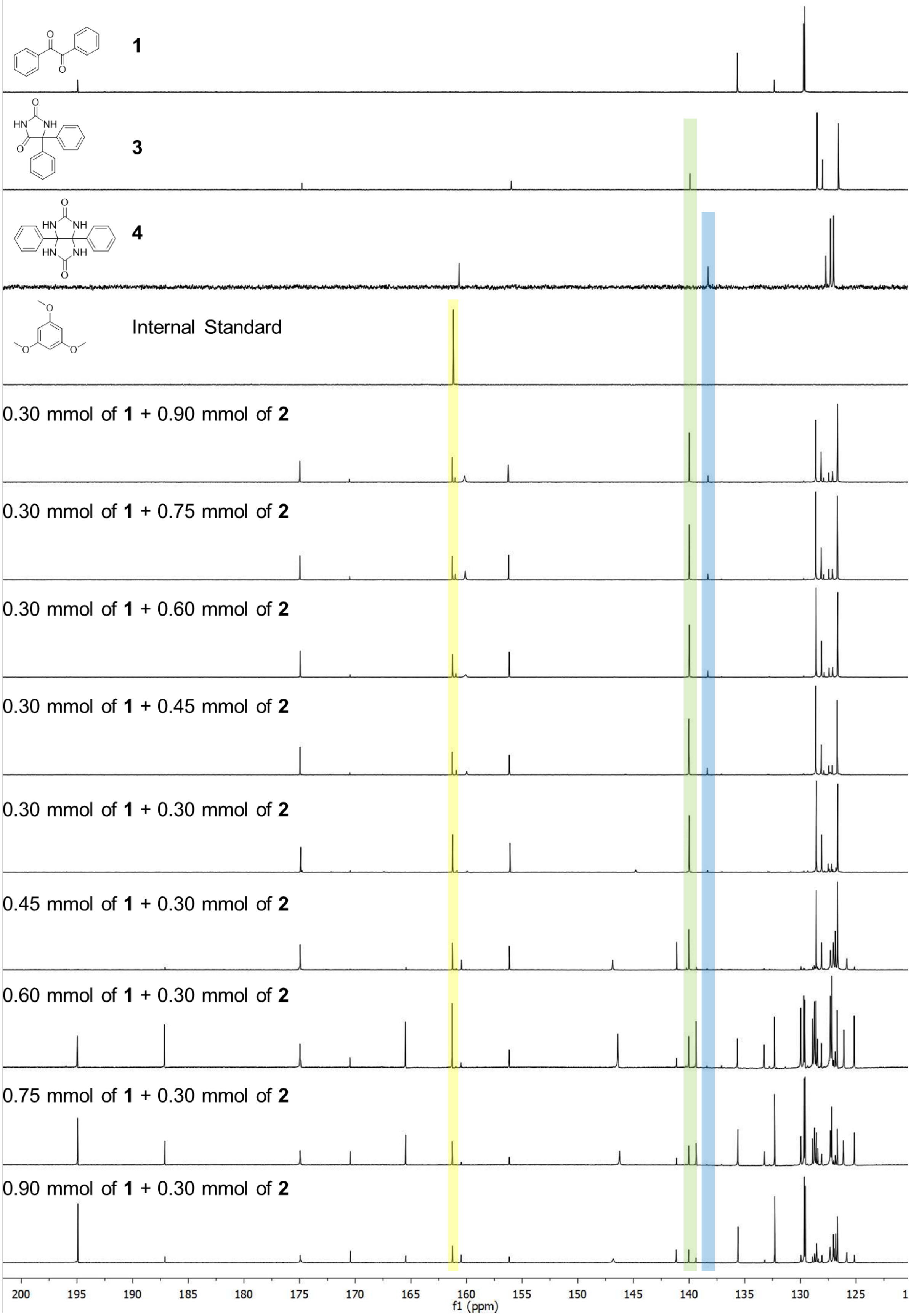


**Figure S12 –** Quantitative $^{13}$C NMR analysis of the extracted reaction mixture for reaction optimization; see Section 1.09 for reaction conditions.

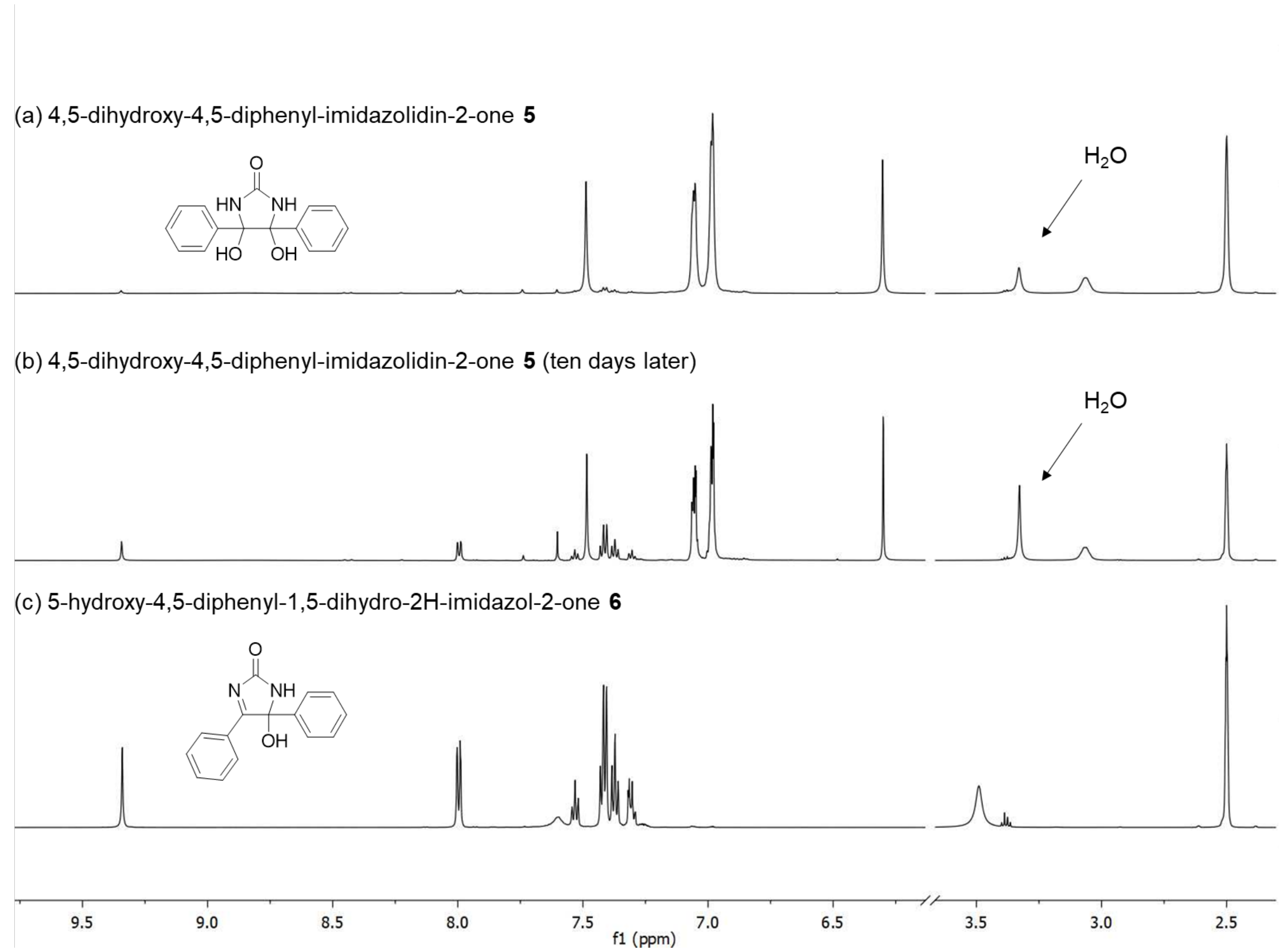


**Figure S13 –** Dehydration of **5** into **6** monitored by $^1$H NMR spectroscopy. The NMR tube initially containing only 4,5-dihydroxy-4,5-diphenyl-imidazolidin-2-one **5** in DMSO-$d_6$-(a) was filled with argon atmosphere and sealed with parafilm. After ten days the same tube was recorded again (b), clearly showing the signals of 5-hydroxy-4,5-diphenyl-1,5-dihydro-2*H*-imidazol-2-one **6** (c), together with an increased intensity for the peak of dissolved water at 3.33 ppm.

## 3. Experimental synthetic procedures

### 3.1 Phenytoin (3)

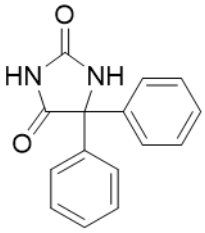

Reaction conditions

1. Take a 15 mL PMMA milling jar and equip it with one 10 mm stainless steel (weighing 4.0 g) milling ball.
2. In open air, add in sequence to the milling jar: benzil (**1**) (126.0 mg, 0.60 mmol), urea (**2**) (36.0 mg, 0.60 mmol) and KOH (67.3 mg, 1.20 mmol).
3. Mill the reaction mixture at 30 Hz for 90 minutes.

Extraction and purification

4. Open the jar, add $NH_4Cl$ (134.0 mg, 2.50 mmol) and 600 mg of silica gel.
5. Mill the mixture at 20 Hz for 20 minutes.
6. Open the jar and recover the reaction mixture.
7. Deposit the recovered solid mixture directly on to a chromatographic column ($SiO_2$) and elute with the appropriate solvent mixture (*n*-hexane:AcOEt 1:1 *v*/*v* or *n*-pentane:AcOEt 2:1 *v*/*v*).
8. Phenytoin (**3**) (114.8 mg, 0.46 mmol) is obtained as a white powder in 73% yield.

Analytics

**Rf** = 0.6 ($SiO_2$, *n*-pentane:AcOEt 1.5:1 *v*/*v*), UV-active (254 nm), visible with $KMnO_4$ or vanillin stain.

**m.p.:** 299.2–300.5 °C (lit.[6b] 293–298 °C).

**$^1$H NMR (600 MHz, DMSO-*d*$_6$):** $\delta$ = 11.10 (br s, 1H, N-*H*), 9.30 (br s, 1H, N-*H*), 7.43–7.31 (m, 10H, Ar–*H*) ppm.

**$^{13}$C{$^1$H} NMR (150 MHz, DMSO-*d*$_6$):** $\delta$ = 174.8, 156.0, 139.9 (2C), 128.5 (4C), 128.0 (2C), 126.6 (4C), 70.2 ppm.

**IR (ATR):** $\nu$ = 3478, 3265, 3196, 2708, 1767, 1717, 1492, 1448, 1399, 1236, 1192, 1014, 984, 745, 695 $cm^{-1}$.

**MS (100 eV, CI):** *m/z* (%): 254 (18) $[M+H]^+$, 253 (100) $[M]^+$.

**MS (70 eV, EI):** *m/z* (%): 253 (20) $[M+H]^+$, 252 (98) $[M]^+$, 209 (47), 180 (100), 104 (34), 77 (27).

The analytical data closely match the ones previously reported in the literature.[6]

### 3.2 3a,6a-Diphenylglycoluril (4)

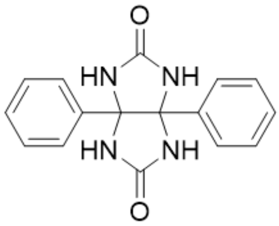

The synthesis of 3a,6a-diphenylglycoluril (**4**) was carried out according to a previously reported protocol.[7]

Reaction conditions
1. Take a 100 mL round bottom flask.
2. Add in sequence: benzil (**1**) (1.0512 g, 5.00 mmol), urea (**2**) (1.2120 g, 20.00 mmol) and KOH (0.1683 g, 3.00 mmol).
3. Add 20.0 mL of 95% EtOH.
4. Place the flask in a sonication bath (Bandelin SONOREX Super RK 106).
5. Irradiate the mixture for 2 hours. *Note*: take care that the temperature of the bath does not exceed 26 °C by constantly exchanging the water.
6. Monitor the reaction by TLC ($SiO_2$, *n*-pentane:AcOEt 1:1 *v*/*v*).

Extraction and purification
7. Remove the solvent using rotary evaporation.
8. Add deionized water to the solid mixture (ca. 30 mL).
9. Filter the white suspension under suction using a sintered glass filter.
10. Wash the solid with water (ca. 30 mL) and ice cold distilled $Et_2O$ (ca. 50 mL).
11. Transfer the remaining powder to a round bottom flask and dry under high vacuum.
12. 3a,6a-Diphenylglycoluril (**4**) (1.0124 g, 3.44 mmol) is obtained as a white powder in 69% yield.

Analytics

**Rf** = 0.3 ($SiO_2$, *n*-pentane:AcOEt 1:1 *v*/*v*), UV-active (254 nm), visible with $KMnO_4$ or vanillin stain.

**m.p.:** 348.7–349.4 °C (lit.[6b] 373–378 °C).

**$^1$H NMR (600 MHz, DMSO-$d_6$):** $\delta$ = 7.73 (s, 4H, N-*H*), 7.11–6.99 (m, 10H, Ar-*H*) ppm.

**$^{13}$C{$^1$H} NMR (150 MHz, DMSO-$d_6$):** $\delta$ = 160.7 (2C), 138.3 (2C), 127.7 (2C), 127.3 (4C), 127.0 (4C), 81.7 (2C) ppm.

**IR (ATR):** $\nu$ = 3231, 2845, 2158, 1672, 1489, 1449, 1224, 1139, 1077, 1029, 949, 770, 692 $cm^{-1}$.

**MS (70 eV, EI):** *m/z* (%): 295 (1) $[M+H]^+$, 294 (1) $[M]^+$, 210 (19), 105 (100), 83 (25), 77 (48), 51 (27).

The analytical data closely match the ones previously reported in the literature.[6b,7]

### 3.3 4,5-Diphenyl-4-imidazolin-2-one (S1)

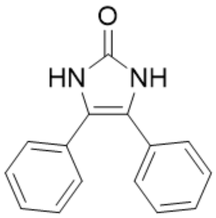

The synthesis of 4,5-diphenyl-4-imidazolin-2-one (**S1**) was carried out according to a previously reported protocol.[8]

Reaction conditions

1. Take a 500 mL round bottom flask and equip it with a stirring bar.
2. Add in sequence: benzoin (24.8 g, 117.0 mmol) and urea (18.0 g, 300.0 mmol).
3. Add 200 mL of ethylene glycol.
4. Equip the reaction flask with a reflux condenser.
5. Heat the reaction mixture at 180 °C in an oil bath for 2 hours under stirring (upon heating the suspension turns to a dark yellow solution).

Extraction and purification

6. Turn off the heating source and reduce the stirring speed to the minimum.
7. Let the reaction mixture to cool to room temperature.
8. Filter the formed precipitate under suction.
9. Wash the yellowish solid in the filter with ice cold distilled $Et_2O$ (ca. 130 mL).
10. Transfer the crude solid product to a 250 mL round bottom flask and dry under high vacuum for 30 minutes.
11. Add 100 mL of 95% EtOH.
12. Heat the suspension at 85 °C for 1 h under vigorous stirring.
13. Turned off the heating source and reduce the stirring speed to the minimum.
14. Once at 30 °C, filter the suspension under suction using a sintered glass filter.
15. Wash the solid in the filter with ice cold 95% EtOH (ca. 100 mL).
16. Transfer the remaining powder to a round bottom flask and dry under high vacuum overnight.
17. 4,5-Diphenyl-4-imidazolin-2-one (**S1**) (15.6 g, 66.0 mmol) is obtained as a white powder in 56% yield.

Analytics

**Rf** = 0.3 ($SiO_2$, *n*-pentane:AcOEt 1:2 *v*/*v*), strongly UV-active (254 nm), visible with $KMnO_4$ or vanillin stain.

**m.p.:** 328.0–329.2 °C (lit.[8] >287 °C).

**$^1$H NMR (400 MHz, DMSO-$d_6$):** $\delta$ = 10.62 (s, 2H, N-*H*), 7.43–7.15 (m, 10H, Ar-*H*) ppm.

**$^{13}$C{$^1$H} NMR (101 MHz, DMSO-$d_6$):** $\delta$ = 154.2, 130.3 (2C), 128.6 (4C), 127.3 (2C), 127.0 (4C), 118.0 (2C) ppm.

**IR (ATR):** $\nu$ = 3142, 3025, 2801, 2330, 1670, 1505, 1447, 1382, 1158, 1071, 1041, 912, 818, 786, 757, 687 $cm^{-1}$.

**MS (100 eV, CI):** *m/z* (%): 237 (100) $[M+H]^+$, 211 (16), 122 (31) .

**MS (70 eV, EI):** *m/z* (%): 237 (8) $[M+H]^+$, 236 (46) $[M]^+$, 235 (2) $[M-H]^+$, 23 (1) $[M-2H]^+$, 105 (23), 104 (87), 77 (100), 76 (24), 51 (85), 50 (26).

The analytical data closely match the ones previously reported in the literature.[8]

### 3.4 5-Hydroxy-4,5-diphenyl-1,5-dihydro-2*H*-imidazol-2-one (6)

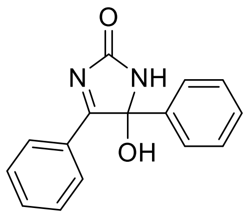

Reaction conditions

1. Take a 50 mL round bottom flask and equip it with a stirring bar.
2. Add 4,5-diphenyl-4-imidazolin-2-one (**S1**) (1000.0 mg, 4.23 mmol).
3. Add 21.0 mL of acetonitrile.
4. Cool the white suspension in an ice bath and stir vigorously.
5. Add aq. $HNO_3$ 62% (1.16 mL, $\rho$ = 1.48 g· $mL^{-1}$, 16.90 mmol) dropwise over 4 minutes (addition speed 0.29 mL/min).
6. Close the reaction flask with a glass cap and allow the suspension to warm up to room temperature by removing the ice bath.
7. After ca. 10 minutes without any visual change, the suspension rapidly turned to a clear yellow solution, from which a white powder copiously starts to precipitate.
8. Stir for additional 2 minutes after the begin of the precipitation.

Extraction and purification

9. Add 5.0 mL of deionized distilled water.
10. Filter the formed precipitate under suction.
11. Wash the solid in the filter with water (ca. 30 mL) to remove any acid residue.
12. Wash the solid in the filter ice cold distilled $Et_2O$ (ca. 20 mL).
13. Transfer the remaining powder to a round bottom flask and dry under high vacuum overnight.
14. 5-Hydroxy-4,5-diphenyl-1,5-dihydro-2*H*-imidazol-2-one (**6**) (880.0 mg, 3.49 mmol) is obtained as a white powder in 83% yield.

Analytics

**Rf** = 0.4 ($SiO_2$, *n*-pentane:AcOEt 1:1.5 *v*/*v*), UV-active (254 nm), visible with $KMnO_4$ or vanillin stain.

**m.p.:** the product decomposes at 117.0–118.0 °C.

**$^1$H NMR (600 MHz, DMSO-*d*$_6$):** $\delta$ = 9.34 (s, 1H, N-*H*), 8.05–7.96 (m, 2H, Ar*H*), 7.60 (br s, 1H, O*H*), 7.50–7.56 (m, 1H, Ar-*H*), 7.44–7.39 (m, 4H, Ar-*H*), 7.39–7.34 (m, 2H, Ar-*H*), 7.33–7.30 (m, 1H, Ar-*H*) ppm.

**$^{13}$C{$^1$H} NMR (150 MHz, DMSO-*d*$_6$):** $\delta$ = 186.9, 165.3, 139.2, 133.1, 129.8 (2C), 128.8, 128.7 (2C), 128.6 (2C), 128.4, 125.0 (2C), 90.7 ppm.

**IR (ATR):** $\nu$ = 3391, 3247, 3061, 1668, 1448, 1408, 1321, 1212, 1126, 1052, 952, 922, 770, 730, 693 $cm^{-1}$.

**MS (100 eV, CI):** *m/z* (%): 253 (4) $[M+H]^+$.

**MS (70 eV, EI):** *m/z* (%): 253 (2) $[M+H]^+$, 252 (3) $[M]^+$, 149 (31), 105 (100), 104 (53), 77 (98), 46 (24), 51 (61), 50 (23).

**MS (ESI):** *m/z*: 275 $[M+Na]^+$.

**MS (APCI):** *m/z*: 253 $[M+H]^+$.

**HRMS (ESI):** *m/z* calculated for $C_{15}H_{12}N_2O_2Na$: 275.0791 $[M+Na]^+$; found: 275.0783.

Visual change observed during the transformation of **S1** to **6** after the addition of aq. $HNO_3$ 62%.

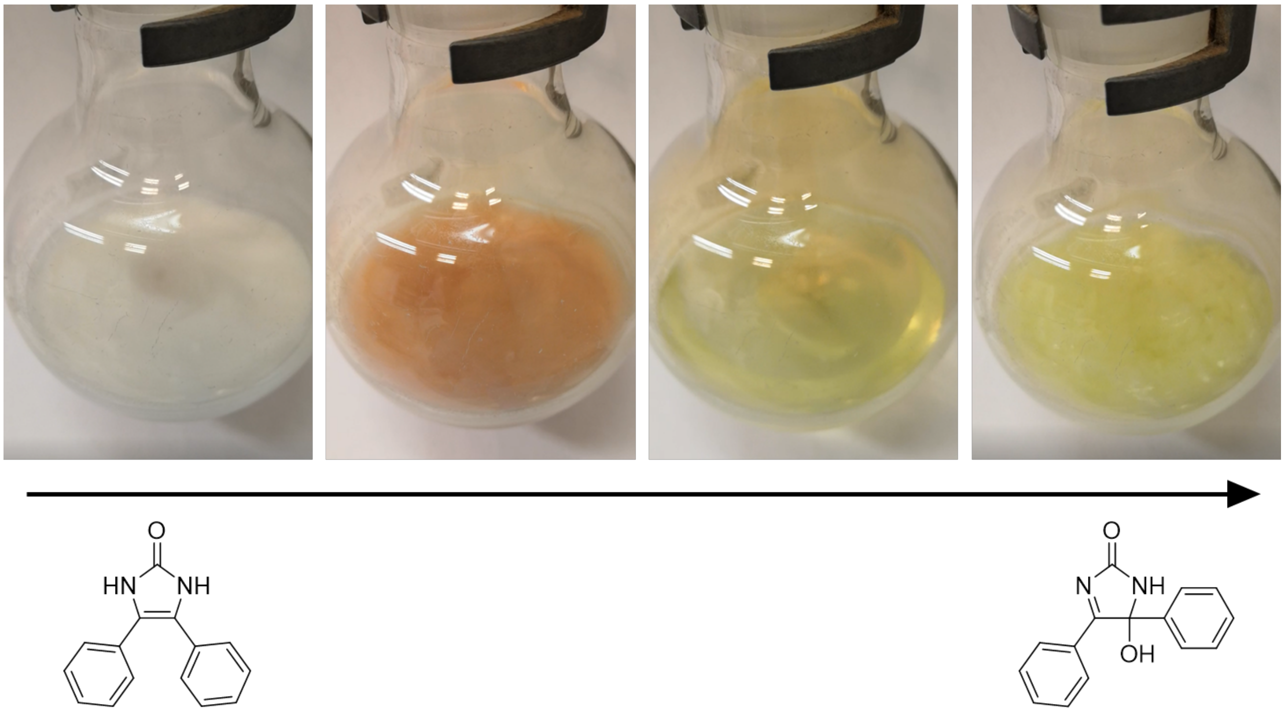

**S1** **6**

### 3.5 4,5-Dihydroxy-4,5-diphenyl-imidazolidin-2-one (5)

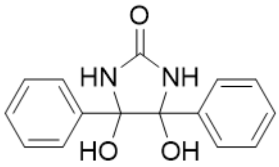

Reaction conditions

1. Take a 100 mL round bottom flask and equip it with a stirring bar.
2. Add 5-hydroxy-4,5-diphenyl-1,5-dihydro-2*H*-imidazol-2-one (**6**) (800.0 mg, 3.17 mmol).
3. Add 33.0 mL of distilled dichloromethane.
4. Add to the white suspension in sequence: triethylamine ($Et_3N$, 93.1 μL, ρ = 0.73 mg·μL$^{-1}$, 0.67 mmol) and deionized water (60.7 μL, 3.37 mmol).
5. Close the reaction flask with a glass cap and seal with parafilm.
6. Stir vigorously at room temperature overnight.

Extraction and purification

7. Evaporate the dichloromethane solvent under vacuum.
8. Add 20 mL of distilled $Et_2O$.
9. Filter the formed precipitate under suction.
10. Wash the solid in the filter with ice cold distilled $Et_2O$ (ca. 20 mL).
11. Transfer the remaining powder to a round bottom flask and dry under high vacuum for 2 hours.
12. 4,5-Dihydroxy-4,5-diphenyl-imidazolidin-2-one (**5**) (716.5 mg, 2.65 mmol) is obtained as a white powder in 84% yield.

Analytics

**m.p.:** 130.5–132.0 °C.

**$^1$H NMR (600 MHz, DMSO-*d*$_6$):** $\delta$ = 7.49 (br s, 2H, N-*H*), 7.94–7.10 (m, 10H, Ar-*H*), 6.30 (br s, 2H, O-*H*) ppm.

**$^{13}$C{$^1$H} NMR (150 MHz, DMSO-*d*$_6$):** $\delta$ = 160.0, 140.7 (2C), 126.8 (2C), 126.6 (4C), 126.4 (4C), 90.4 (2C) ppm.

**IR (ATR):** ν = 3387, 3253, 3063, 2165, 1667, 1447, 1254, 1211, 1125, 1051, 950, 921, 771, 730, 694 cm$^{-1}$.

**MS (70 eV, EI):** *m/z* (%): 253 (1) [M-$H_2O$+H]$^+$, 210 (1), 105 (58), 77 (66), 51 (100), 50 (37).

**MS (100 eV, CI):** *m/z* (%): 271 (3) [M+H]$^+$, 211 (100).

**MS (ESI):** *m/z*: 293 [M+Na]$^+$.

**HRMS (ESI):** *m/z* calculated for $C_{15}H_{14}N_2O_3Na$: 293.0897 [M+Na]$^+$; found: 293.0903.

### 3.6 2-Benzoyl-2,3a,6a-triphenyltetrahydro-5*H*-[1,3]dioxolo[4,5-*d*]imidazol-5-one (7)

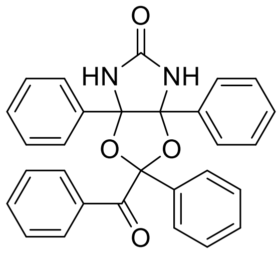

Reaction conditions

1. Take a 15 mL PMMA milling jar and equip it with one 10 mm stainless steel (weighing 4.0 g) milling ball.
2. In open air, add in sequence to the milling jar: benzil (**1**) (252.0 mg, 1.20 mmol) urea (**2**) (72.1 mg, 1.20 mmol) and KOH (33.7 mg, 0.60 mmol).
3. Mill the reaction mixture at 30 Hz for 90 minutes.

Extraction and purification

4. Open the jar, add $NH_4Cl$ (42.8 mg, 0.80 mmol) and 400 mg of silica gel.
5. Mill the mixture at 30 Hz for 20 minutes.
6. Open the jar and recover the reaction crude mixture.
7. Deposit the recovered solid mixture directly from the jar on to a chromatographic column ($SiO_2$) and elute with the appropriate solvent mixture ($SiO_2$, *n*-pentane:AcOEt 2:1 to 1:2 *v*/*v*).
8. After collection of the mixed fractions containing the not separable **6** and **7**, evaporate the solvent to obtain a white powder.
9. Transfer the powder to a 100 mL round bottom flask.
10. Add 50 mL of AcOEt to dissolve the solid.
11. Add 300 mg of silica gel.
12. Evaporate the AcOEt solvent to get a free flowing powder
13. Deposit the solid mixture directly on to a chromatographic column ($SiO_2$) and elute with the appropriate solvent mixture ($SiO_2$, dichloromethane:acetone 7:1 *v*/*v*).
14. After collection of the fractions and evaporation of the solvent, the product 2-benzoyl-2,3a,6a-triphenyltetrahydro-5*H*-[1,3]dioxolo[4,5-*d*]imidazol-5-one (**7**) (10.8 mg, 0.02 mmol) is obtained as a white powder in 4% yield.

Analytics

**Rf** = 0.5 (*n*-pentane:AcOEt 1.5:1 *v*/*v*), UV-active (254 nm), visible with $KMnO_4$ or vanillin stain.

**m.p.:** 212.3–213.1 °C.

**$^1$H NMR (600 MHz, DMSO-*d*$_6$):** $\delta$ = 8.38 (br s, 2H, N*H*), 8.08–8.01 (m, 2H, Ar*H*), 7.83–7.77 (m, 2H, Ar*H*), 7.58–7.54 (m, 2H, Ar*H*), 7.54–7.51 (m, 1H, Ar*H*), 7.51–7.47 (m, 1H, Ar*H*), 7.45–7.39 (m, 2H, Ar*H*), 7.16–7.03 (m, 10H, Ar*H*) ppm.

**$^{13}$C{$^1$H} NMR (150 MHz, DMSO-*d*$_6$):** $\delta$ = 195.0, 158.6, 136.5 (2C), 136.1, 133.9, 132.8, 129.9 (2C), 129.7, 129.2 (2C), 128.4 (2C), 127.5 (5C), 126.8 (5C), 125.5 (2C), 108.3, 100.0 (2C) ppm.

**IR (ATR):** $\nu$ = 3331, 3176, 3108, 2923, 2853, 2324, 1718, 1559, 1448, 1415, 1386, 1231, 1155, 1103, 1028, 976, 941, 894, 776, 725, 690 $cm^{-1}$.

**MS (ESI):** *m/z*: 485 $[M+Na]^+$.

**MS (APCI):** *m/z*: 463 $[M+H]^+$.

**HRMS (ESI):** *m/z* calculated for $C_{29}H_{22}N_2O_4Na$: 485.1472 $[M+Na]^+$; found: 485.1462.

### 3.7 Phenytoin potassium salt (3-K)

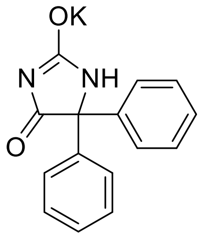


The synthesis of phenytoin potassium salt (**3**-K) was carried out according to a modification of a previously reported protocol.[9]

Reaction conditions

1. Take a 100 mL round bottom flask and equip it with a stirring bar.
2. Add phenytoin (**3**) (5.0 g, 19.8 mmol).
3. Add 20 mL of absolute ethanol and stir to obtain a suspension.
4. Heat the reaction mixture in an oil bath at 42 °C.
5. Under vigorous stirring, add a solution of potassium hydroxide (KOH, 1756.0 mg. 31.3 mmol) in 9 mL of absolute ethanol.
6. Stir the reaction mixture for 13 minutes.

Extraction and purification

7. Turn off the heating source and allow the reaction mixture to cool to room temperature.
8. Add 18 mL of ice-cold *n*-hexane to the reaction flask.
9. Cool the reaction mixture for 20 minutes by placing the reaction flask in an ice bath.
10. Filter the precipitate under suction.
11. Wash the white deliquescent solid several times with ice cold *n*-hexane.
12. Transfer the crude solid product to a 50 mL round bottom flask.
13. Dry overnight under high vacuum at 70 °C.
14. Phenytoin potassium salt (**3**-K), (3130.0 mg, 10.8 mmol) is obtained as a white powder in 54% yield.

Analytics

**m.p.:** 336.2–337.6 °C.

**$^1$H NMR (600 MHz, $D_2O$):** $\delta$ = 7.63–7.16 (m, 10H, Ar*H*) ppm.

**$^{13}$C{$^1$H} NMR (150 MHz, $D_2O$):** $\delta$ = 192.0, 173.8, 140.5 (2C), 128.6 (4C), 128.0 (2C), 127.1 (4C) ppm.

**IR (ATR):** $\nu$ = 3585, 3215, 3062, 2323, 1684, 1582, 1490, 1446, 1360, 1273, 1063, 791, 759, 697 cm$^{-1}$.

**MS (70 eV, EI):** *m/z* (%): 253 (15) [M-K+H]$^+$, 252 (80) [M-K]$^+$, 249 (55), 223 (40), 209 (55), 208 (31), 181 (29), 180 (100), 91 (30), 77 (28).

**MS (ESI):** *m/z*: 313 [M+Na]$^+$.

**HRMS (ESI):** *m/z* calculated for $C_{15}H_{11}N_2O_2K_2$: 329.0089 [M+K]$^+$; found: 329.0085.

### 3.8 Potassium ureate (A)

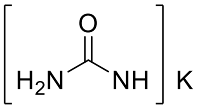

The synthesis of potassium ureate (**A**) was carried out according to a previously reported protocol.[10]

Reaction conditions

1. Take a 250 mL three-neck round bottom flask and equip it with a stirring bar, an isobaric dropping funnel (central neck), a condenser for reduced pressure distillations (with thermometer) and a stopper.
2. Add 60 mL of *o*-xilene.
3. Heat the *o*-xilene in an oil bath at 120 °C for 30 minutes under stirring.
4. Dissolve urea (**2**, 5.0 g, 82.8 mmol) in a potassium methoxide 25% w/w solution in methanol (27.3 m mL $\rho$ = 0.85 mg·$\mu L^{-1}$, 82.8 mmol).
5. Exchange the stopper on the three necks flask with a tubing adaptor and keep the system under a gentle flux of $N_2$.
6. Charge the dropping funnel with the urea/MeOK solution in methanol.
7. Under $N_2$ flux, add the urea/MeOK solution dropwise (over ca. 15 minutes) to the hot *o*-xylene under vigorous stirring. The thermometer on the head of the distillation condenser registers 35-40 °C (MeOH vapors) and methanol condense at the end of it.
8. Increase the oil bath temperature to 125 °C until the temperature at the head of the distillation condenser is dropping, indicating the complete evaporation of methanol (ca. 30 minutes).

Extraction and purification

9. Exchange the $N_2$ gas inlet and the isobaric dropping funnel with glass stoppers.
10. Distill the *o*-xilene under reduced pressure at 135°C.
11. Flush the system with Ar and exchange the distillation condenser with a tubing adaptor.
12. Dry overnight under high vacuum at 70 °C.
13. Transfer the flask to an argon glove box to recover the solid.
14. Potassium ureate (**A**) (8.0 g, 81.1 mmol) is obtained as a white powder in 98% yield and is stored in an argon glove box at room temperature, as it is strongly deliquescent in normal atmosphere.

Analytics

**$^1$H NMR (600 MHz, DMSO-*d*$_6$):** $\delta$ = 3.85 (s, 5H, N-*H*) ppm.

**$^{13}$C{$^1$H} NMR (150 MHz, DMSO-*d*$_6$):** $\delta$ = 160.5 ppm.

# 4. Additional control reactions

### 4.1 Reaction of oven dried benzil (1), urea (2), and KOH under inert argon atmosphere

Samples of benzil, urea and potassium hydroxide were dried in a vacuum oven for two days at 80 °C and afterwards directly transferred to an argon glove box. Following the procedure reported in Section 3.1, a mixture of benzil (**1**) (126.0 mg, 0.60 mmol), urea (**2**) (36.0 mg, 0.60 mmol), and KOH (67.3 mg, 1.20 mmol) was loaded into the milling jar inside a glove box. The jar was closed tight, and the mixture milled at 30 Hz for 90 minutes. After the extraction and purification by column chromatography, phenytoin (**3**) (114.8 mg, 0.46 mmol) was obtained in 76% yield.

### 4.2 Reaction of benzil (1), urea (2), KOH, and $H_2O$

Following the procedure reported in Section 3.1 of the present document, a mixture of benzil (**1**) (126.0 mg, 0.60 mmol), urea (**2**) (36.0 mg, 0.60 mmol), KOH (67.3 mg, 1.20 mmol) and distilled water (21.6 μL $\rho$ = 1.00 mg·μL$^{-1}$, 1.20 mmol) was milled at 30 Hz for 90 minutes. After the extraction and purification by column chromatography, phenytoin (**3**) (90.9 mg, 0.36 mmol) was obtained in 60% yield.

### 4.3 Reaction of 4,5-dihydroxy-4,5-diphenyl-imidazolidin-2-one (5) + KOH

Following the procedure reported in Section 1.9 of the present document, a mixture of 4,5-dihydroxy-4,5-diphenyl-imidazolidin-2-one (**5**) (81.1 mg, 0.30 mmol) and KOH (33.7 mg, 0.60 mmol) was loaded into the milling jar inside a glove box. The jar was closed tight, and the mixture milled at 30 Hz for 90 minutes. After the extraction, the product mixture was analyzed by $^{13}$C NMR in DMSO-*d*$_6$, revealing a full conversion of the starting material into phenytoin (**3**).

### 4.4 Reaction of 5-hydroxy-4,5-diphenyl-1,5-dihydro-2*H*-imidazol-2-one (6) + KOH

Following the procedure reported in Section 3.1 of the present document, a mixture of 5-hydroxy-4,5-diphenyl-1,5-dihydro-2H-imidazol-2-one (**6**) (75.7 mg, 0.30 mmol) and KOH (33.7 mg, 0.60 mmol) was loaded into the milling jar inside a glove box. The jar was closed tight, and the mixture milled at 30 Hz for 90 minutes. After the extraction, the product mixture was analyzed by $^{13}$C NMR in DMSO-*d*$_6$, revealing a full conversion of the starting material into phenytoin (**3**).

### 4.5 Reaction of 5-hydroxy-4,5-diphenyl-1,5-dihydro-2*H*-imidazol-2-one (6) + urea (2) + KOH

Following the procedure reported in Section 3.1 of the present document, a mixture of 5-hydroxy-4,5-diphenyl-1,5-dihydro-2H-imidazol-2-one (**6**) (75.7 mg, 0.30 mmol) and urea (**2**) (90.1 mg, 1.50 mmol) was loaded into the milling jar inside a glove box together with KOH (5.61 mg, 0.10 mmol). The jar was closed tight, and the mixture milled at 30 Hz for 90 minutes. After the extraction, the product mixtures were analyzed by $^{13}$C NMR in DMSO-*d*$_6$, revealing an incomplete conversion of **6** together with the formation of product 3a,6a-diphenylglycoluril (**4**).

### 4.6 Reaction of 5-hydroxy-4,5-diphenyl-1,5-dihydro-2H-imidazol-2-one (6) + benzil (1) + KOH

Following the procedure reported in Section 3.1 of the present document, a mixture of 5-hydroxy-4,5-diphenyl-1,5-dihydro-2*H*-imidazol-2-one (**6**) (75.7 mg, 0.30 mmol) and benzil (**1**) (315.0 mg, 1.50 mmol) was loaded into the milling jar inside a glove box together with KOH (5.61 mg, 0.10 mmol). The jar was closed tight, and the mixture milled at 30 Hz for 90 minutes. After the extraction, the product mixtures were analyzed by $^{13}$C NMR in DMSO-*d*$_6$, revealing an uncomplete conversion of **6** together with the formation of 2-benzoyl-2,3a,6a-triphenyltetrahydro-5*H*-[1,3]dioxolo[4,5-*d*]imidazol-5-one (**7**).

### 4.7 Variations from the reaction conditions reported in Section 3.6

In order to exclude any interference of the extraction procedure in the mechanochemical formation of products 5-hydroxy-4,5-diphenyl-1,5-dihydro-2*H*-imidazol-2-one (**6**) and 2-benzoyl-2,3a,6a-triphenyltetrahydro-5*H*-[1,3]dioxolo[4,5-*d*]imidazol-5-one (**7**), the reaction was repeated varying the conditions reported in Section 3.6 of the present document. The results obtained are shown below.

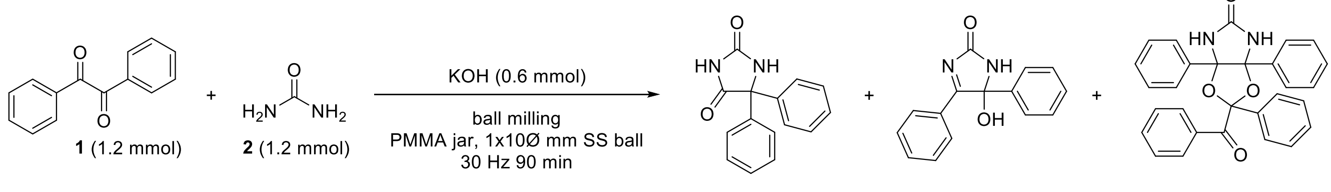


3 6 7

| Entry | Variations from the reported conditions | Result |
|---|---|---|
| **1** | - | Unreacted **1** and **2**, formation of **3**, **6** and **7** (Section 3.6) |
| **2** | *No KOH* | **1** only |
| **3** | *No KOH; $NH_4Cl$ since the beginning* | **1** only |
| **4** | *No KOH; No **2;** $NH_4Cl$ since the beginning* | **1** only |
| **5** | *No $NH_4Cl$* | see Entry 1 |
| **6** | *No $SiO_2$* | see Entry 1 |
| **7** | *Sand instead of $SiO_2$* | see Entry 1 |
| **8** | *EtOH instead of $SiO_2$* | see Entry 1 |

## 5. References


[1] Mestrelab Research S.L. *MestReNova*, **2018**, Version 12.0.1-20560.

[2] G. R. Fulmer, A. J. M. Miller, N. H. Sherden, H. E. Gottlieb, A. Nudelman, B. M. Stoltz, J. E. Bercaw, K. I. Goldberg, K. I. *Organometallics* **2019**, *29*, 2176–2179.

[3] S. Lukin, K. Užarević, I. Halasz, *Nat. Protoc*. **2021**, *16*, 3492–3521.

[4] P. H. C. Eilers, H. F. M. Leiden University Medical Center Report, Leiden (**2005**).

[5] P. H. C. Eilers, A Perfect Smoother. *Anal. Chem*. **2003**, *75*, 3631–3636.

[6] a) L. Konnert, B. Reneaud, R. M. de Figueiredo, J.-M. Campagne, F. Lamaty, J. Martinez, E. Colacino, *J. Org. Chem.* **2014**, *79*, 10132–10142; b) I. Boudebouz, S. Arrous, I. V. Parunov, *Russ. J. Org. Chem.* **2019**, *55*, 1874–1877.

[7] J. T. Li, X. R. Liu, M. X. Sun, *Ultrason. Sonochem.* **2010**, *17*, 55–57.

[8] Y. B. Kim, C. S. Kim, C. K. Lee, *J. Heterocycl. Chem.* **1994**, *31*, 1653–1656.

[9] H. Shi, Y. Chen, M. Jiang, K. Shao, D. Yuan, Z. Wen (Ningbo Polytechnic), CN109456271A, **2019**.

[10] J. Park, H. J. Kim, *J. Korean Chem. Soc.* **2016**, *60*, 251–256.

## 6. Copies of the NMR spectra

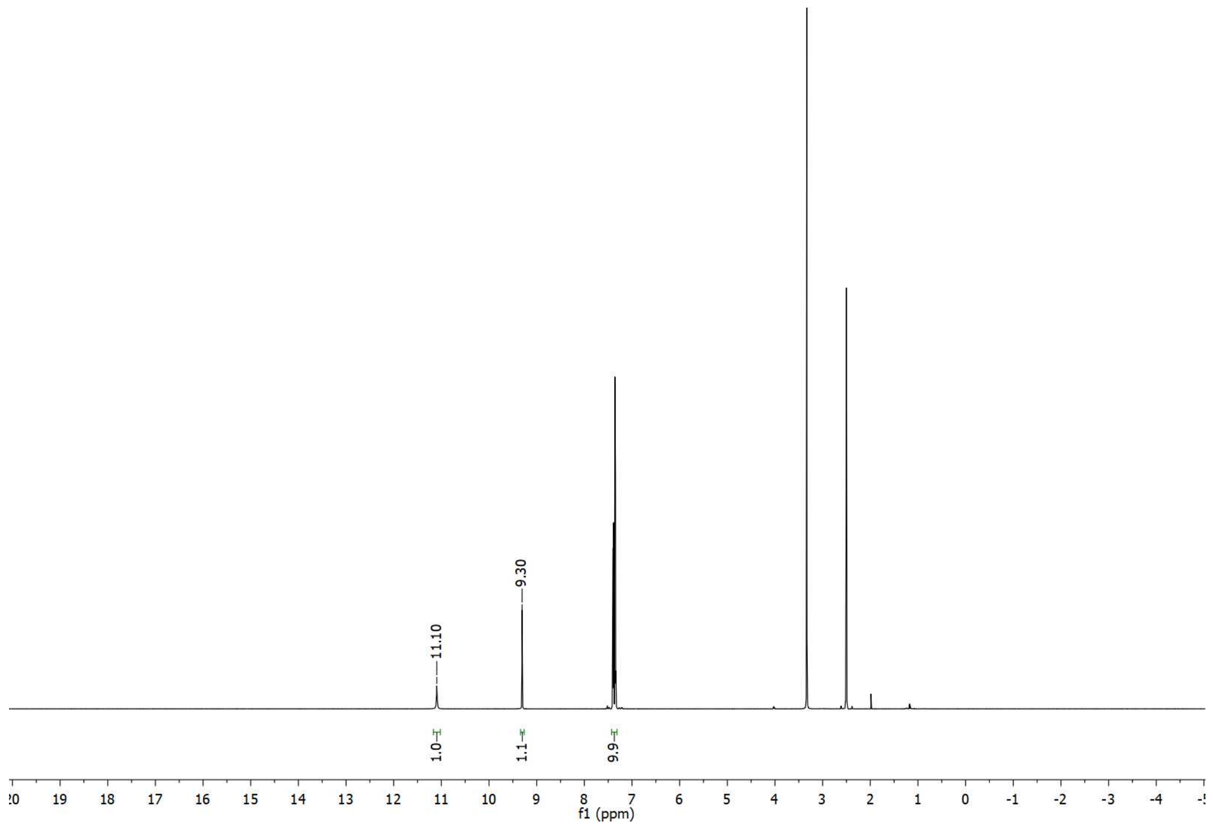


**Figure S14 –** $^{1}H$ NMR (600 MHz, DMSO-$d_6$) spectrum of **3**.

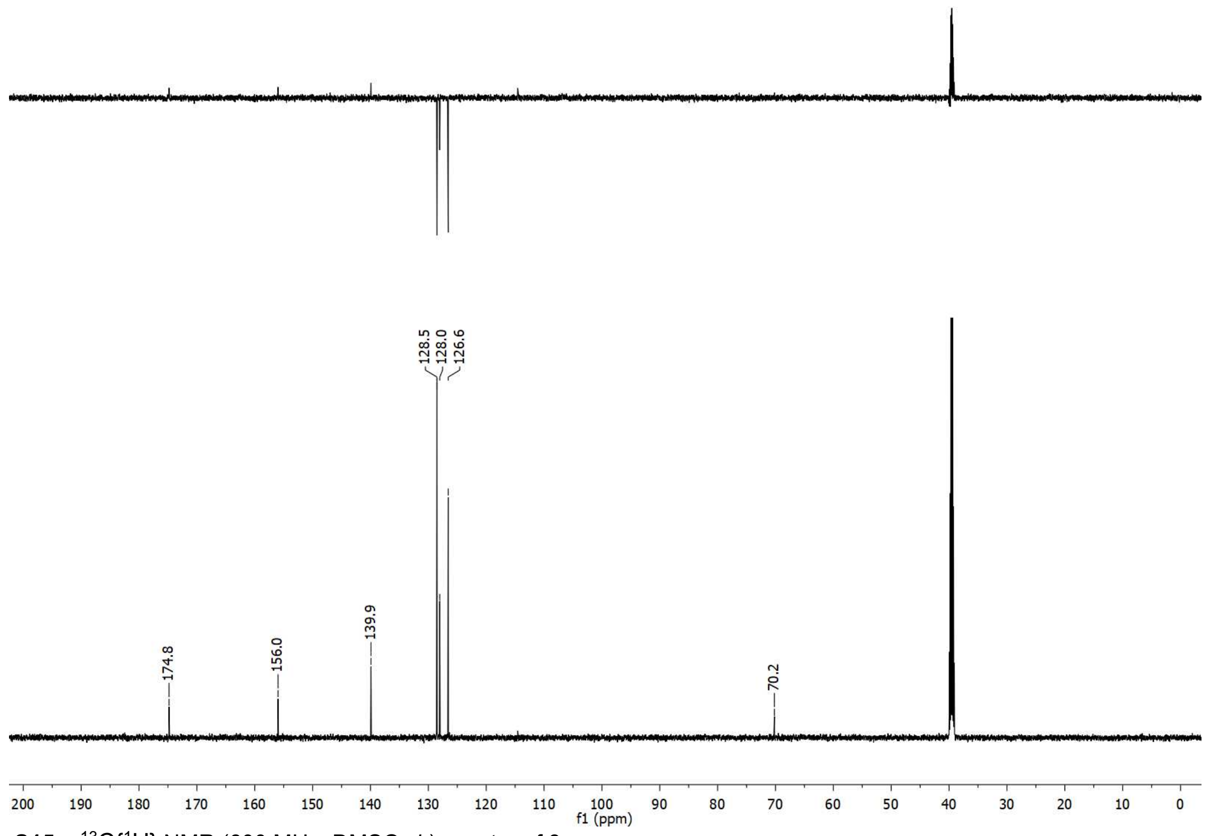


**Figure S15 –** $^{13}C\{^{1}H\}$ NMR (600 MHz, DMSO-$d_6$) spectra of **3**.

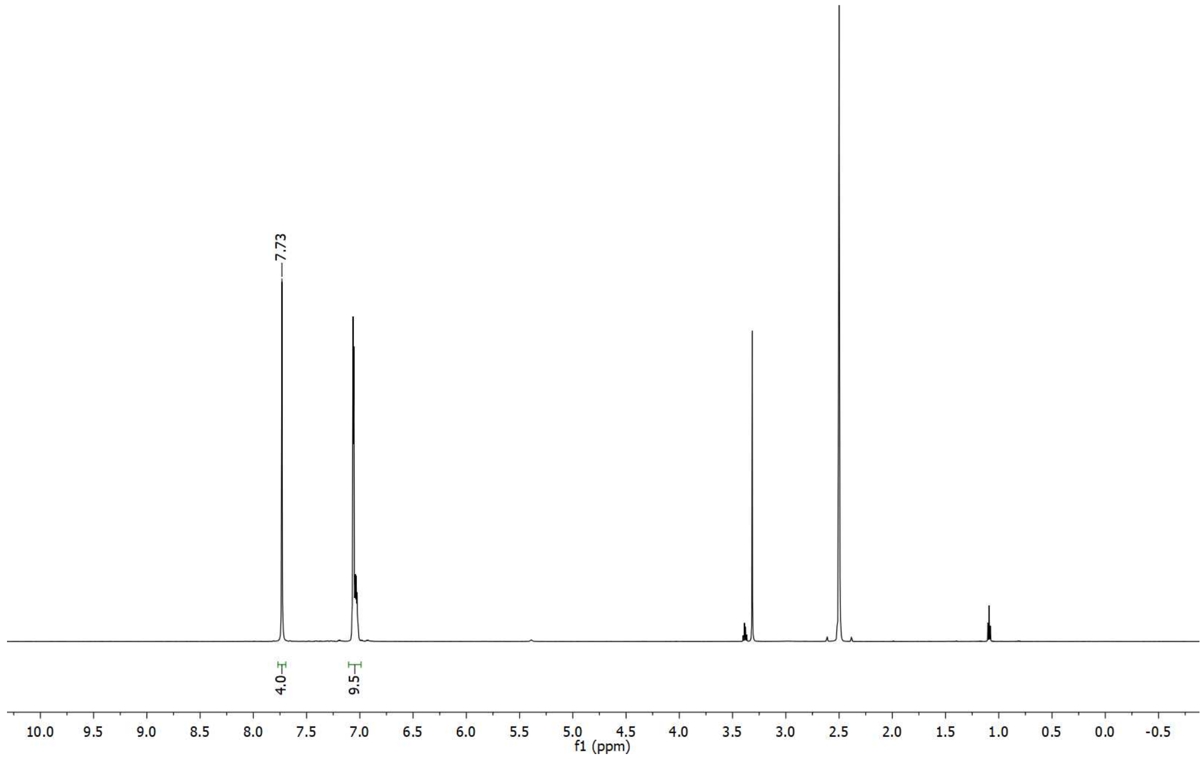


**Figure S16 –** $^{1}H$ NMR (600 MHz, DMSO-$d_6$) spectrum of **4**.

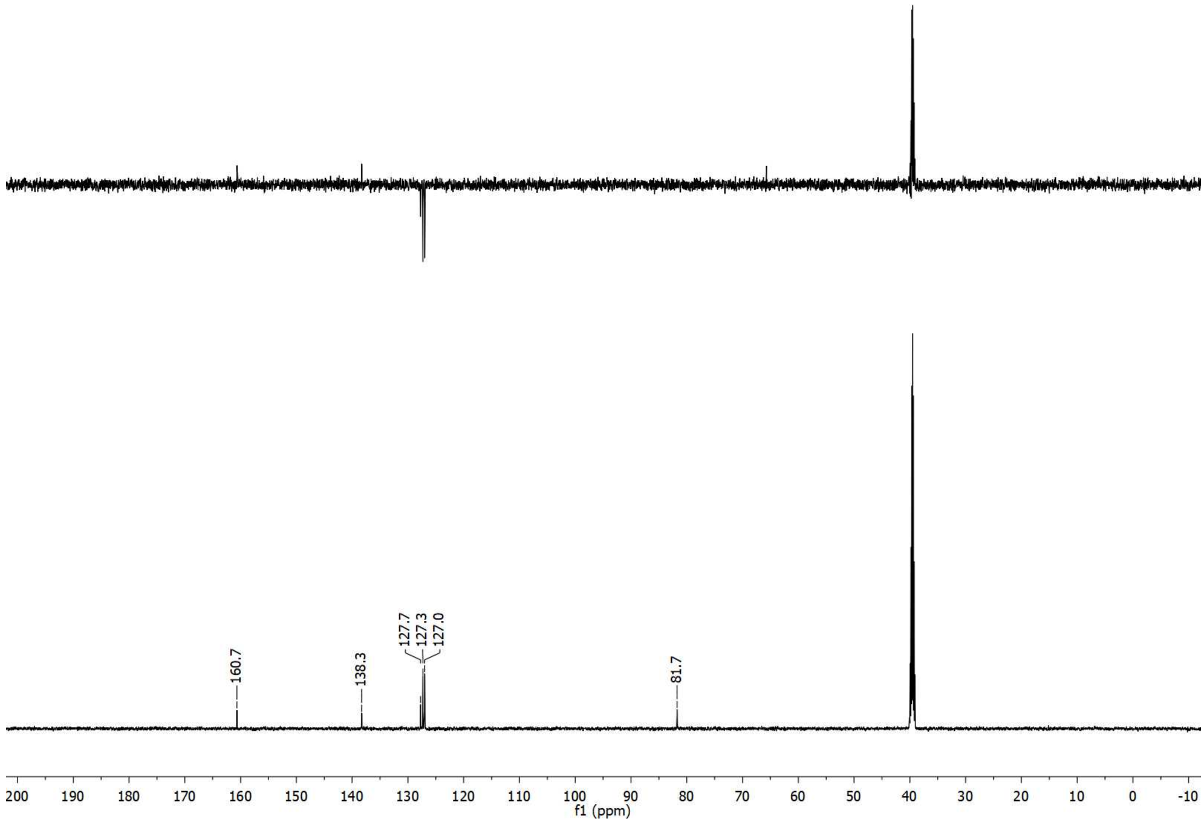


**Figure S17 –** $^{13}C\{^{1}H\}$ NMR (150 MHz, DMSO-$d_6$) spectra of **4**.

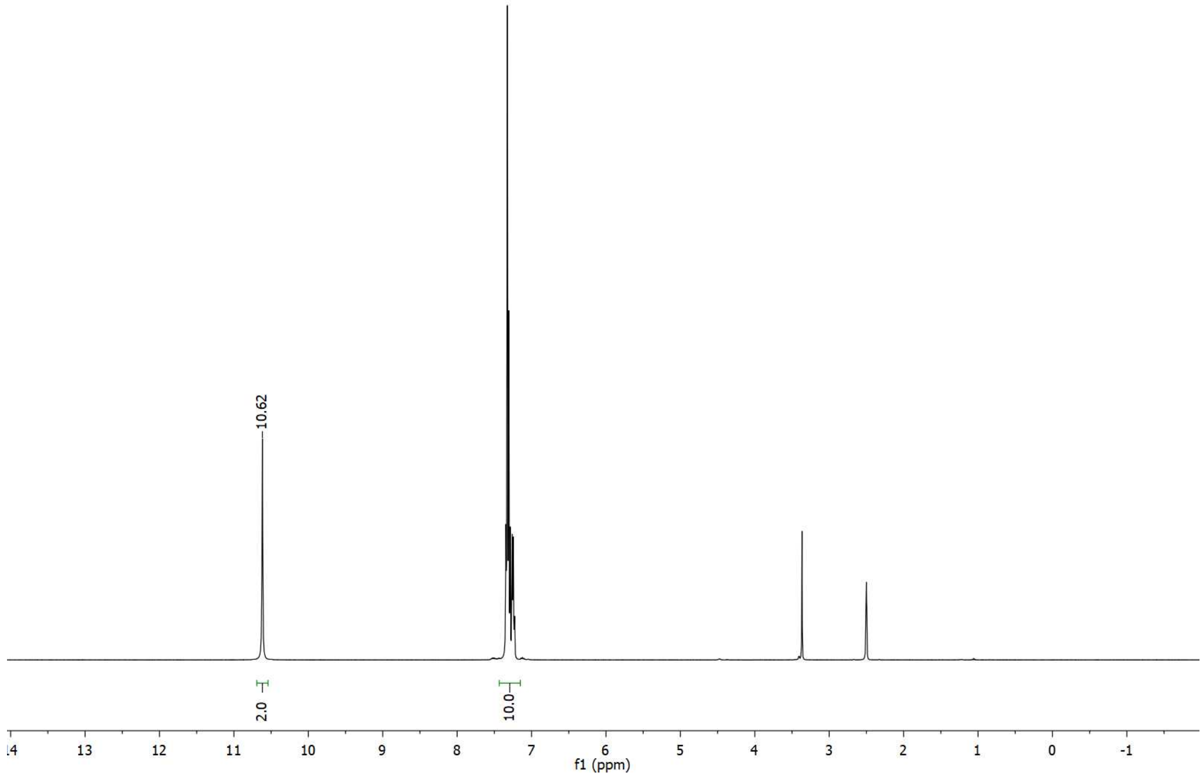


**Figure S18 –** $^{1}H$ NMR (101 MHz, DMSO-$d_6$) spectrum of **S1**.

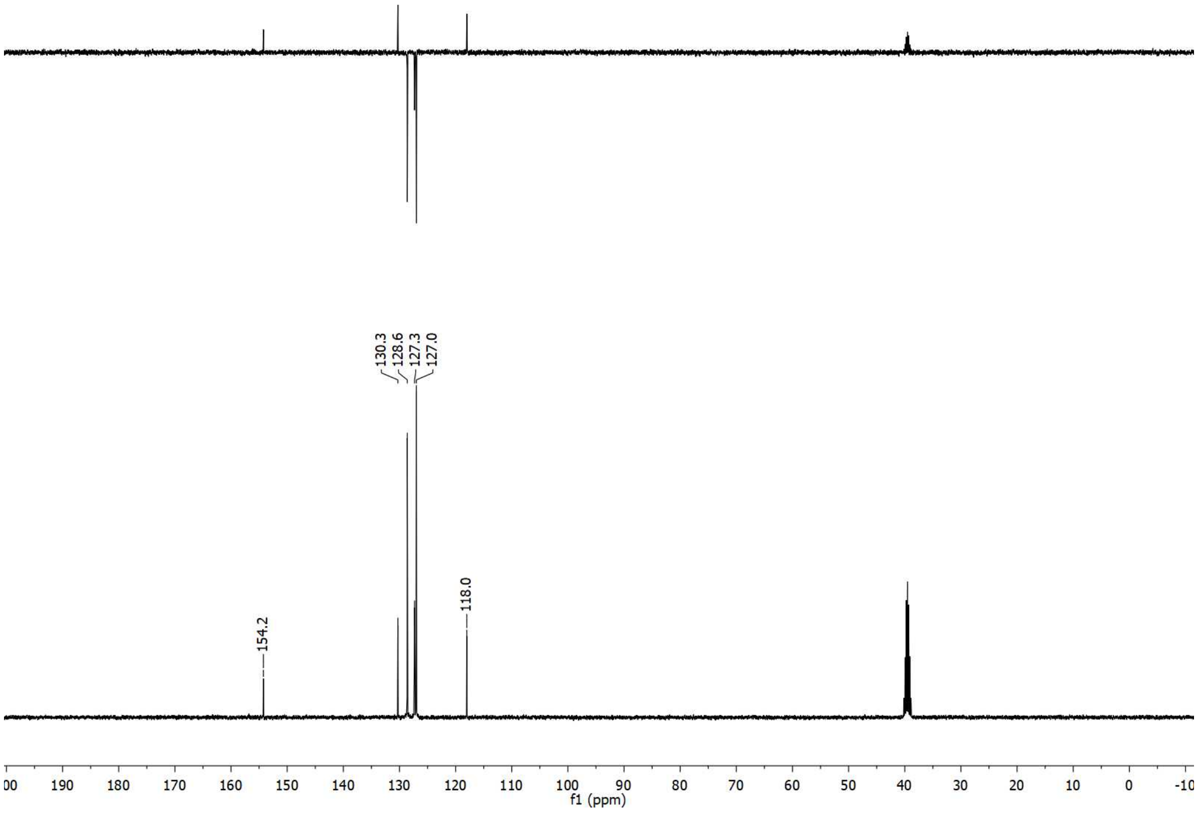


**Figure S19 –** $^{13}C\{^{1}H\}$ NMR (400 MHz, DMSO-$d_6$) spectra of **S1**.

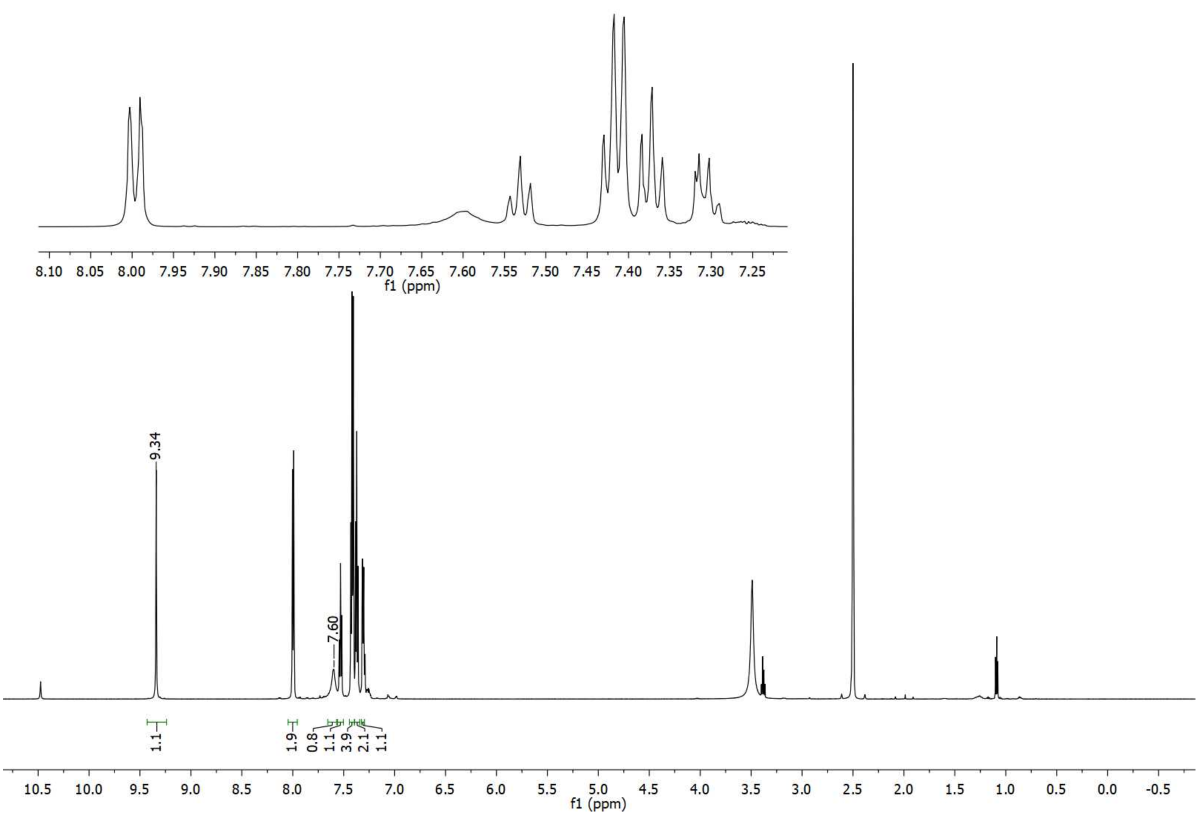


**Figure S20 –** $^{1}$H NMR (600 MHz, DMSO-$d_6$) spectrum of **6**.

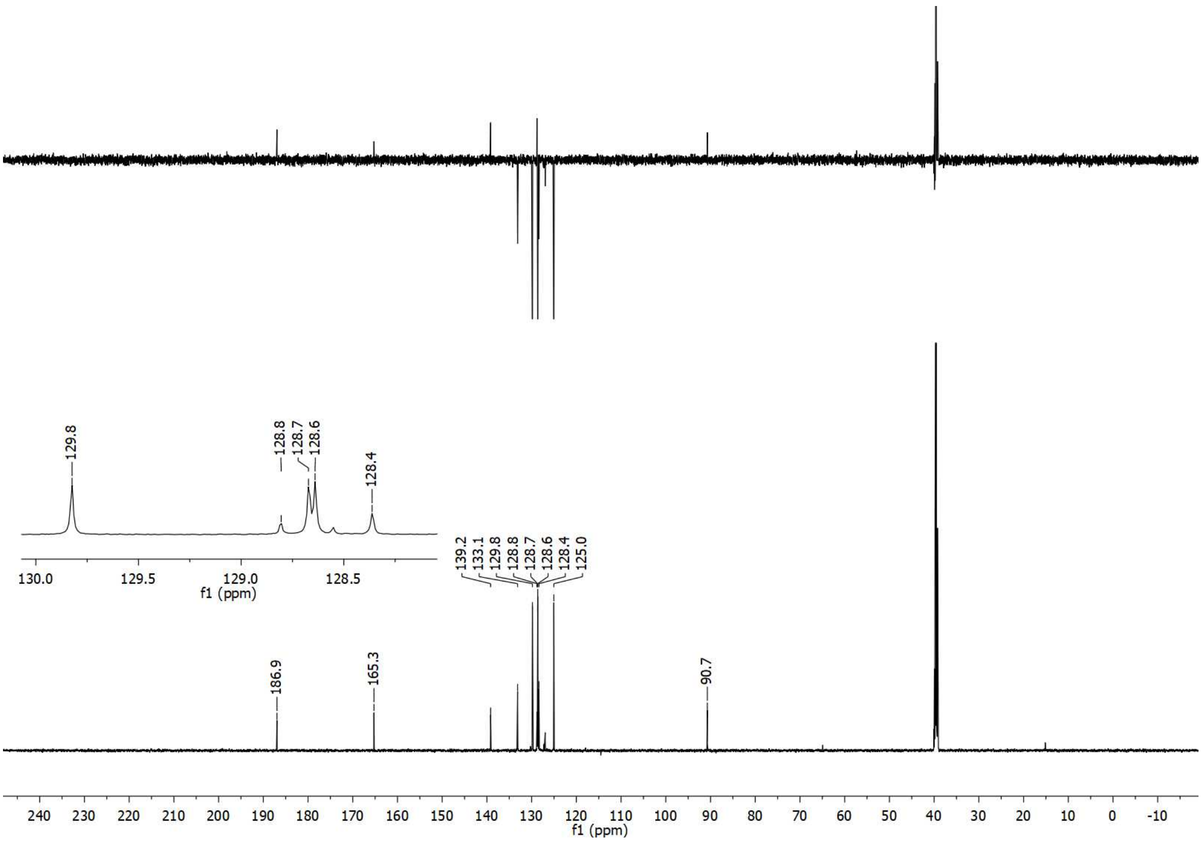


**Figure S21 –** $^{13}C\{^{1}H\}$ NMR (150 MHz, DMSO-$d_6$) spectra of **6**.

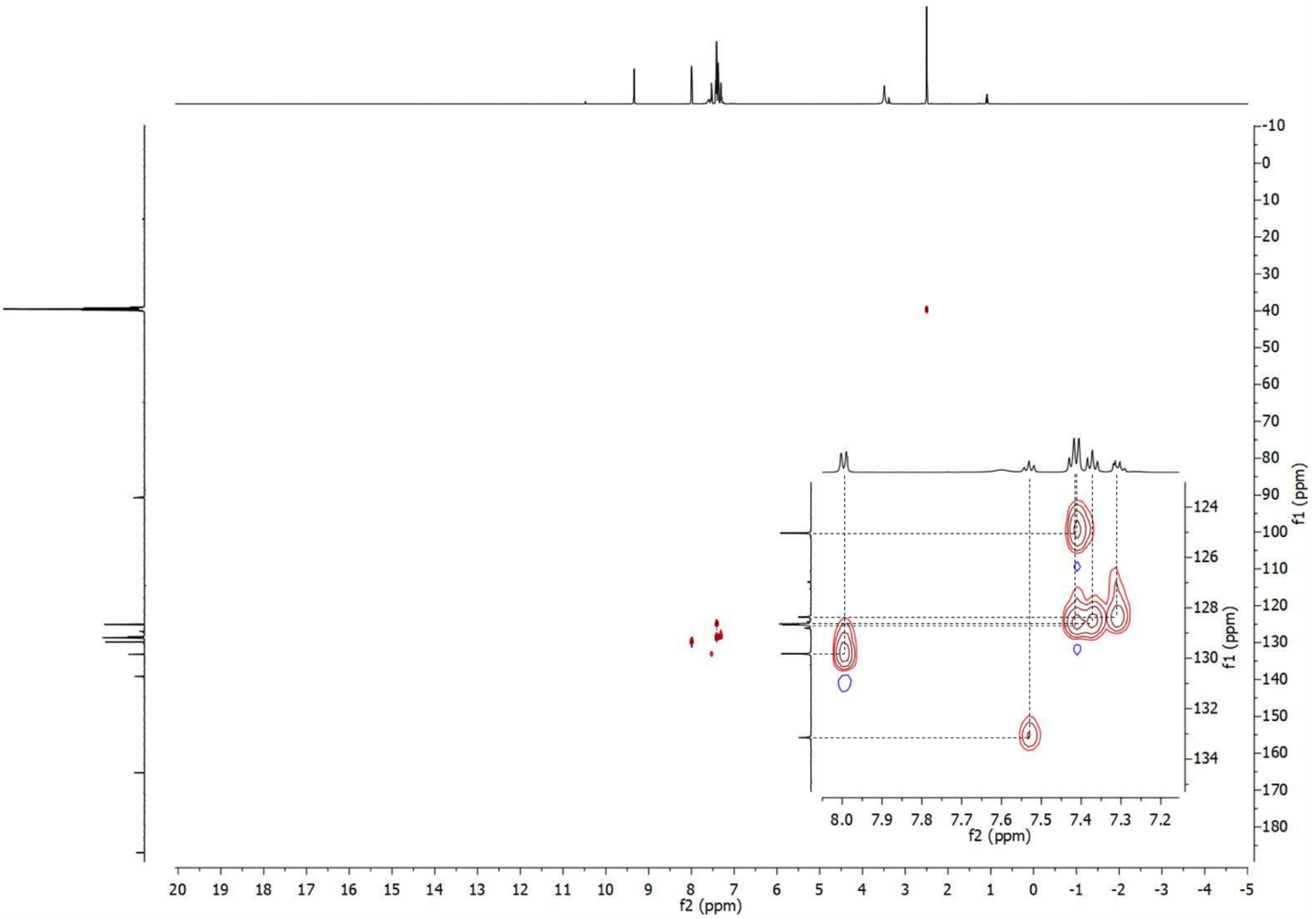


**Figure S22 –** $^{1}H,^{13}C$-HSQC NMR (600,150 MHz, DMSO-$d_6$) spectrum of **6.**

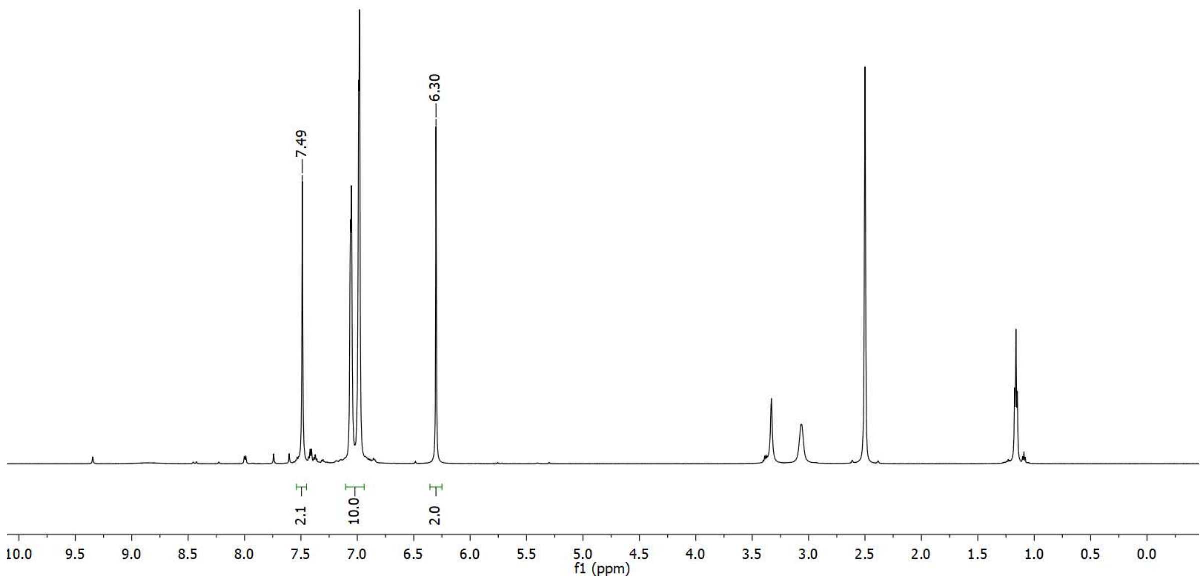


**Figure S23 –** $^{1}$H NMR (600 MHz, DMSO-$d_6$) spectrum of **5.**

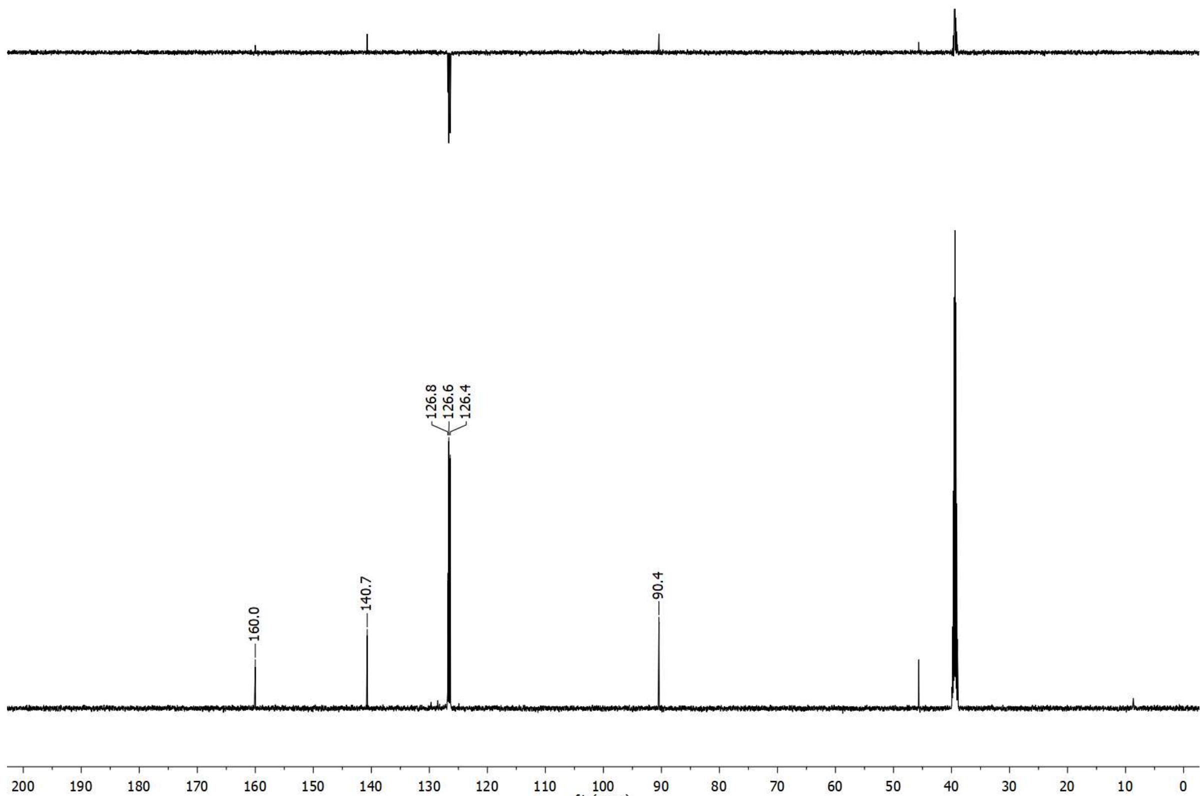


**Figure S24 –** $^{13}C\{^1H\}$ NMR (150 MHz, DMSO-$d_6$) spectra of **5.**

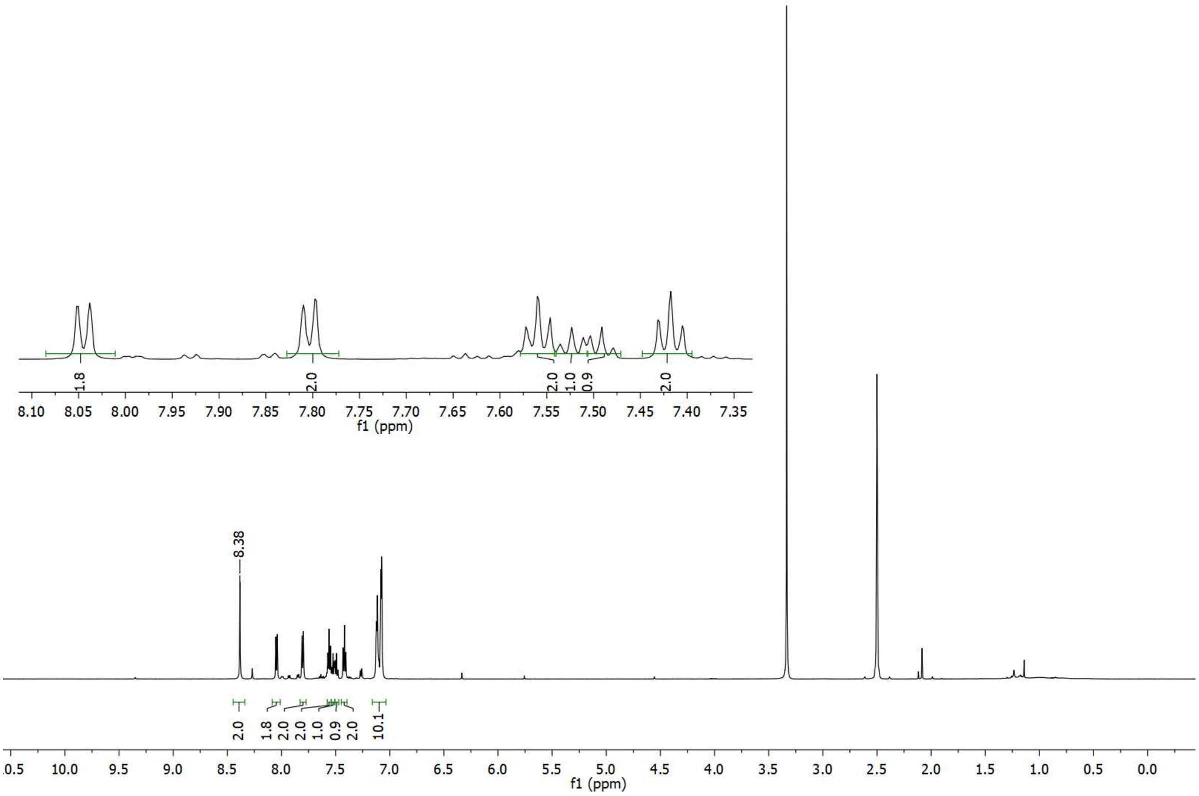

**Figure S25 –** $^{1}$H NMR (600 MHz, DMSO-$d_6$) spectrum of **7**.

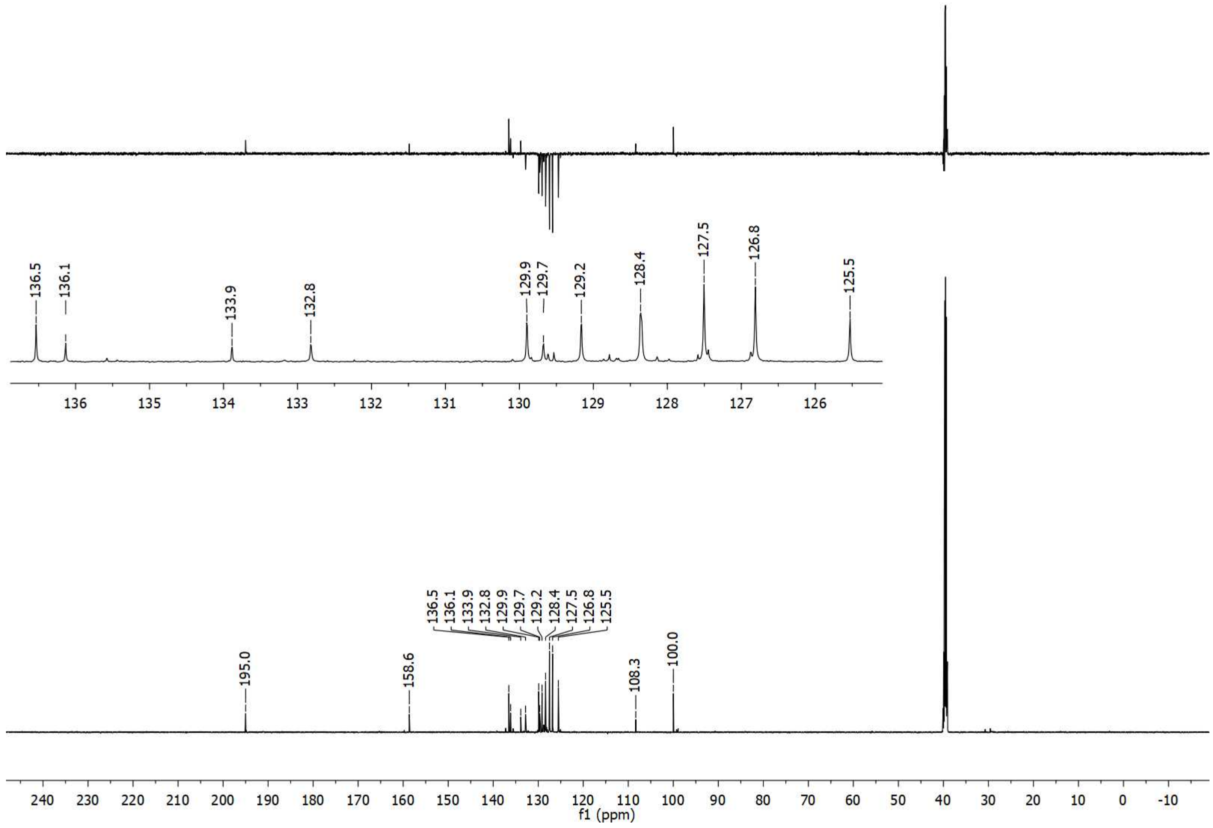

**Figure S26 –** $^{13}$C{$^{1}$H} NMR (150 MHz, DMSO-$d_6$) spectra of **7**.

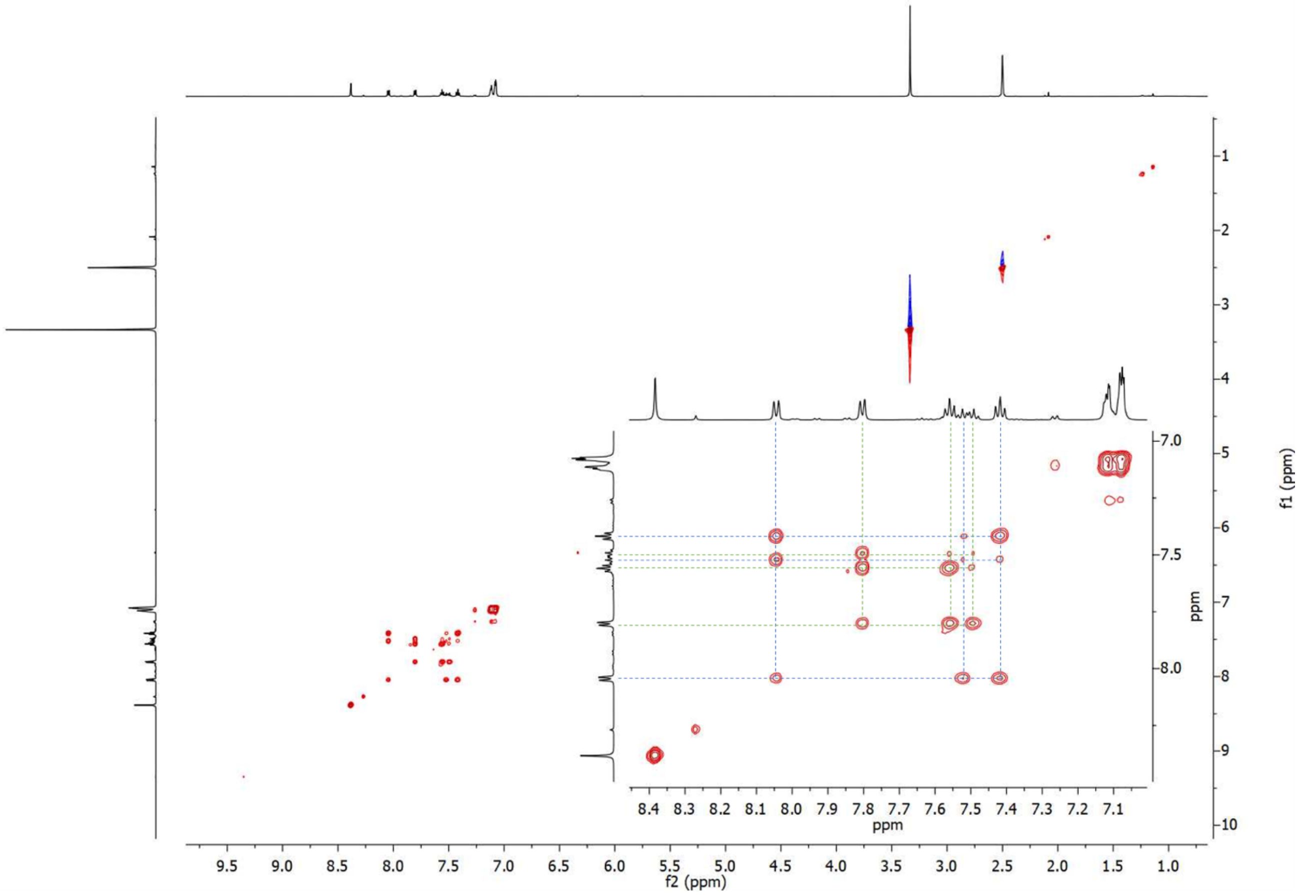


**Figure S27 –** $^{1}$H,$^{1}$H-TOCSY NMR (600,600 MHz, DMSO-$d_6$) spectrum of **7**.

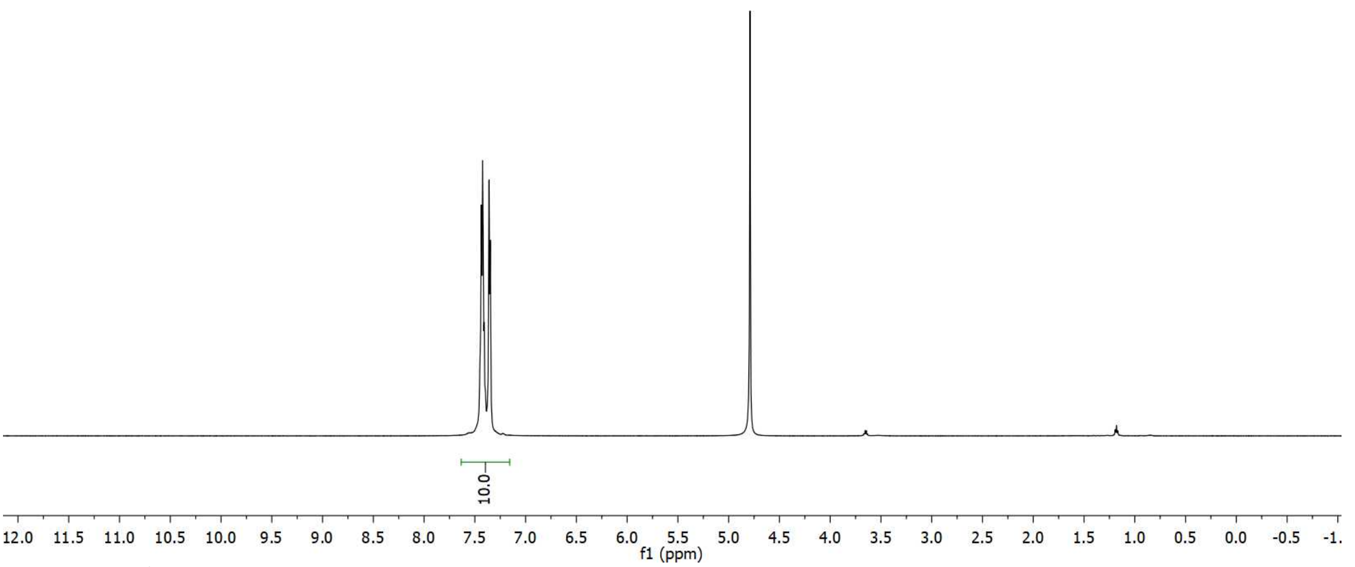


**Figure S28 –** $^{1}H$ NMR (600 MHz, $D_2O$) spectrum of **3**-K.

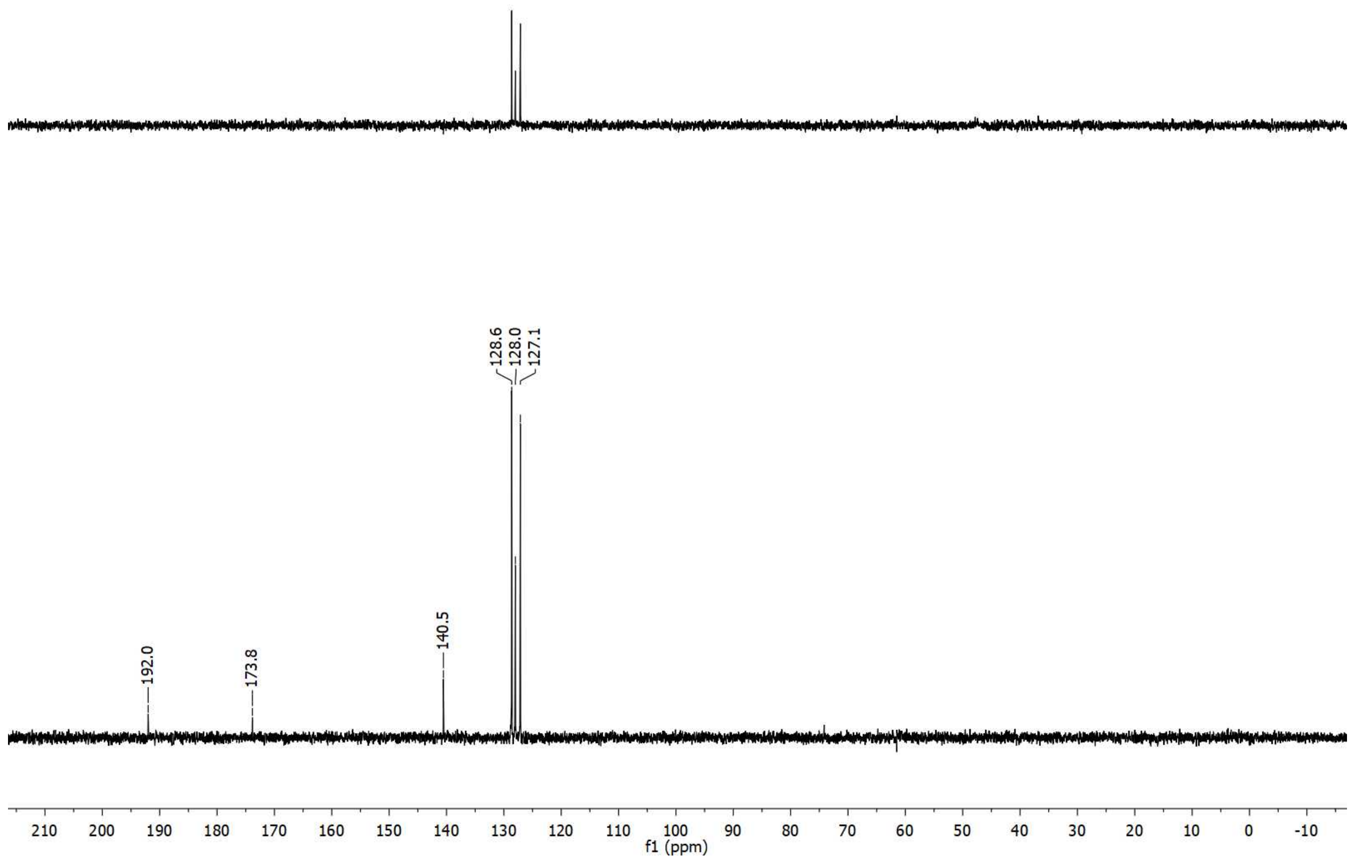


**Figure S29 –** $^{13}C\{^{1}H\}$ NMR (150 MHz, $D_2O$) spectra of **3**-K

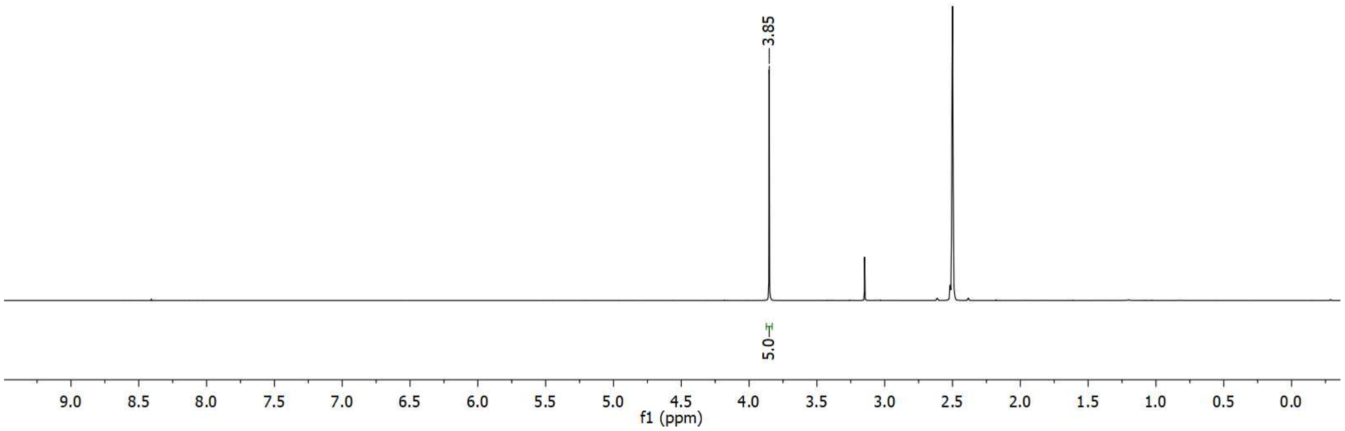


**Figure S30 –** $^1$H NMR (600 MHz, DMSO-$d_6$) spectrum of **A**.

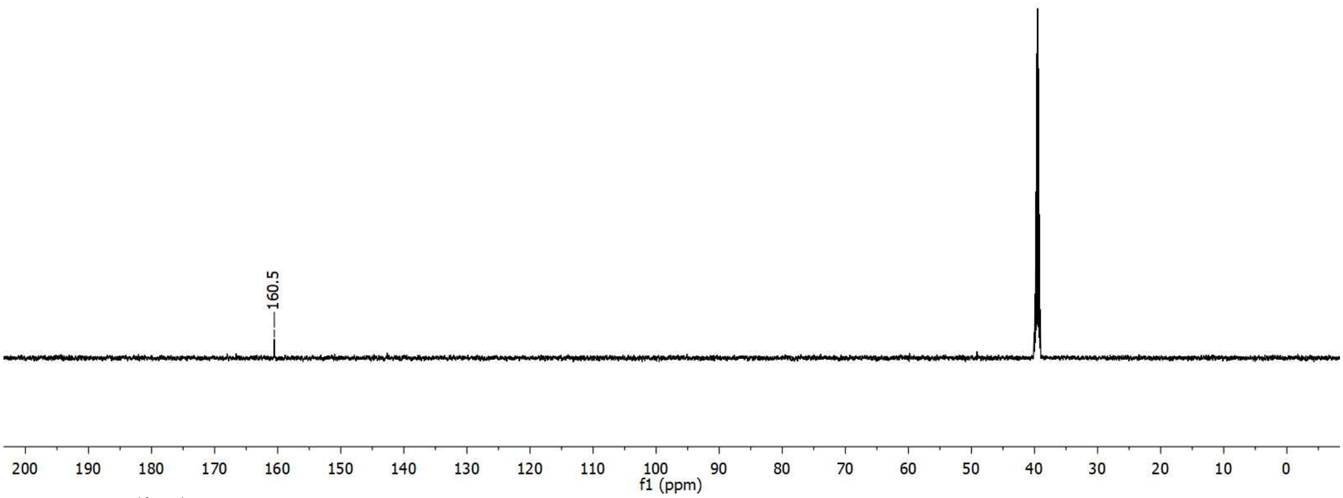


**Figure S31 –** $^{13}$C{$^1$H} NMR (150 MHz, DMSO-$d_6$) spectrum of **A**.
